# Bex1 is essential for ciliogenesis and harbours biomolecular condensate-forming capacity

**DOI:** 10.1186/s12915-022-01246-x

**Published:** 2022-02-10

**Authors:** Emi Hibino, Yusuke Ichiyama, Atsushi Tsukamura, Yosuke Senju, Takao Morimune, Masahito Ohji, Yoshihiro Maruo, Masaki Nishimura, Masaki Mori

**Affiliations:** 1 Molecular Neuroscience Research Centre (MNRC), Shiga University of Medical Science, Seta Tsukinowa-cho, Otsu, Shiga 520-2192 Japan; 2 Graduate School of Pharmaceutical Sciences, Nagoya University, Furo-cho, Chikusa-ku, Nagoya, 464-8601 Japan; 3 Department of Ophthalmology, Shiga University of Medical Science, Seta Tsukinowa-cho, Otsu, Shiga 520-2192 Japan; 4 Department of Paediatrics, Shiga University of Medical Science, Seta Tsukinowa-cho, Otsu, Shiga 520-2192 Japan; 5 Department of Vascular Physiology, National Cerebral and Cardiovascular Centre Research Institute, 6-1 Kishibe-Shimmachi, Suita, Osaka, 564-8565 Japan; 6 Research Institute for Interdisciplinary Science, Okayama University, 3-1-1 Tsushima-naka, Kita-ku, Okayama, 700-8530 Japan

**Keywords:** Intrinsically disordered protein (IDP), Primary cilia, Bex1, Juvenility-associated genes (JAGs), Tubulin polymerization

## Abstract

**Background:**

Primary cilia are sensory organelles crucial for organ development. The pivotal structure of the primary cilia is a microtubule that is generated via tubulin polymerization reaction that occurs in the basal body. It remains to be elucidated how molecules with distinct physicochemical properties contribute to the formation of the primary cilia.

**Results:**

Here we show that *brain expressed X-linked 1* (*Bex1*) plays an essential role in tubulin polymerization and primary cilia formation. The Bex1 protein shows the physicochemical property of being an intrinsically disordered protein (IDP). Bex1 shows cell density-dependent accumulation as a condensate either in nucleoli at a low cell density or at the apical cell surface at a high cell density. The apical Bex1 localizes to the basal body. *Bex1* knockout mice present ciliopathy phenotypes and exhibit ciliary defects in the retina and striatum. Bex1 recombinant protein shows binding capacity to guanosine triphosphate (GTP) and forms the condensate that facilitates tubulin polymerization in the reconstituted system.

**Conclusions:**

Our data reveals that Bex1 plays an essential role for the primary cilia formation through providing the reaction field for the tubulin polymerization.

**Supplementary Information:**

The online version contains supplementary material available at 10.1186/s12915-022-01246-x.

## Background

Juvenile animals possess physiological characteristics distinct from those of adults. These features include growth, maturation, regeneration, and plasticity capabilities, among others. With the aim of revealing the molecular machinery underlying juvenile-predominant capacities, we identified juvenility-associated genes (JAGs) as genes that are selectively highly expressed in juvenile cells [1]. Here, we show the physiological role of *Bex1*, a JAG that is encoded on the X chromosome and predominantly expressed in juvenile organs in mice. The molecular function of Bex1 largely remains to be determined, although its roles have been reported in the contexts of tumorigenesis [2–4], muscle regeneration [5] and the response to heart failure [6].

Cells possess cilia, which are hair-like structures that modulate and sense the extracellular environment. The propeller-like motion or periodic fluttering of motile cilia affects the extracellular space by generating flows or waves of extracellular fluids, thereby determining right-left asymmetry [7], or driving the cerebrospinal fluid flow [8]. Nonmotile cilia, or primary cilia, are found in numerous cell types in vertebrates [9] and sense physical and chemical inputs, thus adjusting the cellular response to external environment [10–13]. The mechanosensing ability of cilia contributes to the determination of tubular diameter in vascular vessels and renal tubules [14]. Their ligand sensing ability is exemplified by a role in Hedgehog signalling [15], which regulates cell growth [16] and causes medulloblastoma when it occurs in excess [17].

The different types of cilia are formed by distinct mechanisms, as implied by the identification of gene mutations that affect ciliogenesis. Ciliopathies can be subdivided into motile and nonmotile ciliopathies based on the major clinical manifestations [18, 19]. Defects in motile cilia are found in primary ciliary dyskinesia and manifest as situs inversus (reversal of left-right asymmetry) and chronic respiratory infections that are due to dyskinetic airway cilia. Dysfunctional primary cilia are found in primary ciliopathies including Joubert syndrome that is characterized by cerebellar hypoplasia, retinal dysplasia and renal cystic disease [20, 21].

The formation of the primary cilium starts with the migration of centrioles to the cell surface. The centrioles contain nine microtubule triplets and mature into a centrosome that is referred to as a basal body in the context of ciliogenesis [22, 23]. Basal body maturation occurs along with the accumulation of components essential for tubulin nucleation to form the molecular condensed region known as the pericentriolar matrix (PCM [24];). The PCM forms the reaction field for tubulin polymerization. The molecular components of the PCM include Pericentrin [25], Cep152/Asl, Cep192/SPD-2, Cep215/Cnn, and SPD-5 but have yet to be completely defined [24, 26]. Tubulin polymerization is mediated by the polymerizing reaction of α-/β-tubulin heterodimers catalysed with guanosine triphosphate (GTP) as the energy source [27]. The basal body is thought to form the reaction field where microtubule synthesis occurs by supplying reaction substrates through a mechanism that has remained elusive thus far.

Cellular compartmentalization is a mechanism that increases the efficiency of molecular reactions. An intriguing feature of intrinsically disordered proteins (IDPs) is their tendency to form subcellular compartments specialized for molecular reactions [28–30]. IDPs lack a stable secondary structure due to biassed amino acid components or repeated sequence elements in intrinsically disordered regions (IDRs). IDPs induce phase separation and form biomolecular condensates such as nucleoli [31]. Concentrating components can increase reaction kinetics, thus enhancing reaction efficiency [31, 32].

In this paper, we describe a role of Bex1 as an IDP that is essential for ciliogenesis both in vitro and in vivo. Bex1 forms a condensate at the basal body of the primary cilium. *Bex1* mutation causes ciliopathy phenotypes in mice. A recombinant Bex1 protein reconstitutes the Bex1 biomolecular condensates that induce the polymerization of tubulin, the pivotal structure of the primary cilium. Our findings elucidate the essential role of Bex1 biomolecular condensates in ciliogenesis.

## Results

### *Bex1* is a JAG essential for cell growth

**Fig. 1:**
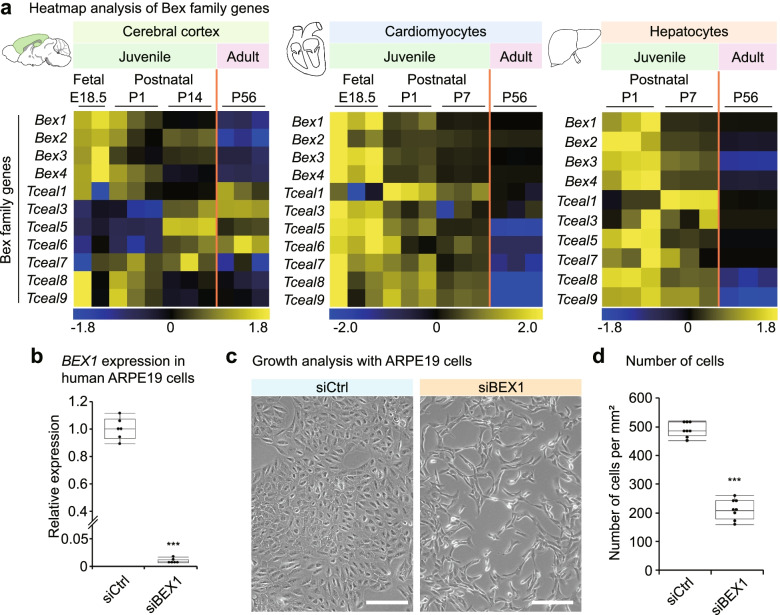
*Bex1* is expressed in juvenile and is essential for cell growth. **a** Heatmap analysis of *Bex* family genes in the cerebral cortex, cardiomyocytes and hepatocytes at different embryonic (E18.5) and postnatal days (P1, P7 and P56). E, embryonic day. P, postnatal day. **b** qPCR analysis of *BEX1* in human ARPE19 cells 48 h after transfection of control siRNA or *BEX1* siRNA. Data were normalized to *TUBB*. **c** Phase contrast images of ARPE19 cells 72 h after transfection of control siRNA or *BEX1* siRNA. Scale bar = 250 μm. **d** Number of cells per field 72 h after transfection with control siRNA or *BEX1* siRNA. ∗∗∗ *p* < 0.001; Student’s *t* test. The data are presented as the means ± standard deviations

To search for genes that are relevant to juvenile growth and tissue maturation, we performed transcriptome analysis and identified the Bex family of genes, whose members are expressed predominantly in juveniles rather than in adults (Fig. 1a and Additional file 1: Fig. S1a). The Bex family consists of 11 genes (*Bex1*, *Bex2*, *Bex3*, *Bex4*, *Tceal1*, *Tceal3*, *Tceal5*, *Tceal6*, *Tceal7*, *Tceal8* and *Tceal9*), many of which showed ubiquitous juvenile-predominant expression in the investigated cerebral cortex tissues, cardiomyocytes and hepatocytes (Fig. 1a and Additional file 1: Fig. S1a). To study their functions in growth, we performed a cellular analysis focusing on *Bex1*, which showed the most restricted expression in juveniles (Additional file 1: Fig. S1a). The depletion of *BEX1* in ARPE19 human retinal pigment epithelial cells (Fig. 1b) resulted in significant impairment of cellular growth (Fig. 1c,d), suggesting a role of *BEX1* in cell growth. To address the cell type and species dependence of this effect, we performed *Bex1* depletion in Neuro2a mouse neural progenitor cells (Additional file 1: Fig. S1b) and confirmed the significant suppression of cellular growth (Additional file 1: Fig. S1c,d). These results indicated the indispensable role of *Bex1* in cell growth and led us to investigate the molecular function of *Bex1*.

### Bex1 has the physical characteristics of an IDP

To investigate the molecular function of Bex1, we performed domain searches in protein databases such as pfam, InterPro, SMART and PROSITE. None of the analyses predicted any functional domains in the Bex1 protein. We then analysed physical properties of Bex1 protein. Structural modelling with Phyre2 [33] showed that Bex1 contained an IDR that occupied 54.0% of its entire length (Additional file 1: Fig. S2a). Long IDRs (> 40% of the entire length) were found in all the Bex family proteins, suggesting that Bex family proteins function as IDPs (Additional file 1: Fig. S2a). Further predictive analyses conducted according to the physical characteristics of Bex family proteins with the Protein DisOrder prediction System (PrDOS) and DISOPRED3 algorithms verified that they contained highly disordered domains predominantly located in the N-terminal half of the proteins (Additional file 1: Fig. S2b).

**Fig. 2:**
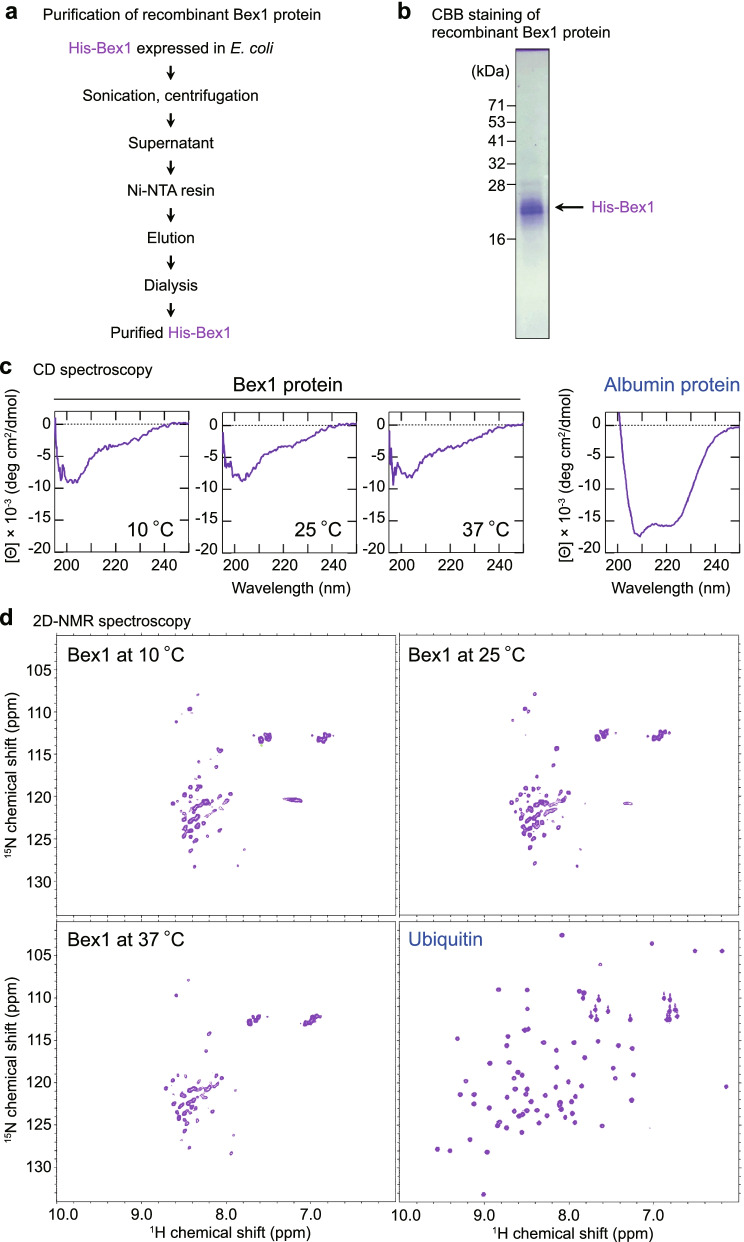
Bex1 has the physical identity as an IDP. **a** The purification scheme to obtain the recombinant Bex1 protein. **b** Coomassie brilliant blue (CBB) staining of purified Bex1 protein. **c** Far-UV circular dichroism (CD) spectrum of the recombinant Bex1 protein at 10, 25 and 37°C. A typical random coil CD spectrum was detected for the Bex1 protein. The measurements of a typical structured protein, albumin, are shown for comparison. Θ, molar ellipticity. **c** Two-dimensional nuclear magnetic resonance (2D-NMR) spectrum of the ^15^N-labelled recombinant Bex1 protein. The ^1^H-^15^N heteronuclear single quantum coherence (HSQC) spectrum was measured at pH 7.3 and 10, 25 and 37°C. The spectrum of a typical structured protein, ubiquitin, are shown for comparison. Signals observed around 6.8–7.6 ppm in Bex1 plots were derived from side-chain amide groups

To investigate the physical properties of the Bex1 protein via physicochemical analyses, we established a purification workflow for a recombinant Bex1 protein without any denaturing step (Fig. 2a,b). A previous attempt to analyse the Bex1 protein included the denaturing steps of boiling and the application of acidic conditions (pH 5.0), which hampered the assessment of the protein under physiological conditions [34]. The native-state Bex1 recombinant protein was assessed under physiological conditions at 37°C. Circular dichroism (CD) spectroscopy with Bex1 revealed a striking pattern that differed from that of albumin, which was investigated as a control structured protein (Fig. 2c). The CD spectrums of Bex1 was similar to an IDR of SP1 that is a well-characterized IDP [35], in that they showed a negative peak at around 200 nm (Additional file 1: Fig. S2c). The results suggested that the Bex1 protein lacked a stable secondary structure such as α-helixes or β-strands (Fig. 2c). As protein structure is known to be affected by temperature, CD spectral analyses were conducted at temperatures of 10, 25 and 37°C that gave similar results, confirming that the Bex1 protein did not have a stable secondary structure (Fig. 2c).

To further investigate the physical properties of the Bex1 protein, two-dimensional nuclear magnetic resonance (2D-NMR) spectroscopy was performed to analyse the tertiary structure of Bex1 under nondenaturing conditions at the different temperature of 10, 25 and 37°C. The Bex1 spectra showed ^1^H chemical shifts in a narrower range (7.8 - 8.5 ppm) than that observed for ubiquitin, a control structured protein (6.5 – 10.0 ppm), suggesting that Bex1 lacked stable tertiary structures (Fig. 2d). The 2D-NMR spectrums of Bex1 were similar to those of an IDR of TAF4 (Additional file 1: Fig. S2d, [36]). The spectral patterns concentrated at approximately 8.0 ppm were characteristic of a protein in a random coil state [37], suggesting that the purified Bex1 protein adopts a random coil state under physiological conditions (Fig. 2d). These analyses provided evidence that Bex1 possesses the physical identity of an IDP under physiological conditions.

### Cell density-dependent localization of BEX1

**Fig. 3:**
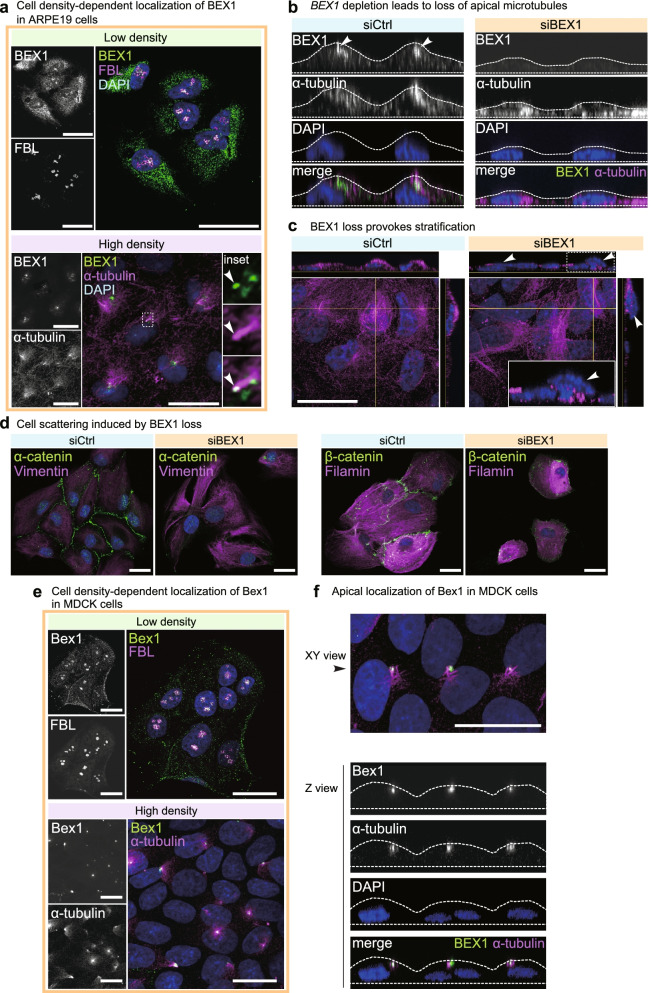
Bex1 localizes to nucleoli and apical side of cells in the cell density-dependent manner. **a** Immunostaining of BEX at different cell densities in ARPE19 cells. At a low cell density, BEX1 localized to nucleoli, which were stained with a fibrillarin (FBL) antibody. At a high cell density, BEX1 showed a condensed pattern that colocalized with α-tubulin. The inset shows the localization of the BEX1 condensate (arrowhead) to tubulin fibres. Nuclei were stained with DAPI. Scale bar = 25 μm. **b** Reconstructed *z*-axis view of ARPE19 cells at the high cell density. In control knockdown cells (siCtrl), the immunostaining showed localization of BEX1 to the apical side of cells where tubulin was enriched (arrowhead). In *BEX1*-depleted cells (siBEX1), the enrichment of tubulin at the apical surface was lost and cells were flattened. **c** Reconstructed *z*-axis view of ARPE19 cells showing the stratification provoked by *BEX1* knockdown. Arrowheads indicate the cells overriding other cells. Scale bar = 25 μm. **d** Cell scattering was induced by *BEX1* knockdown. Immunostaining of cell-cell junction protein α-catenin and β-catenin and cytoskeletal protein Vimentin and Filamin. ARPE19 cells were transfected with control siRNA or *BEX1* siRNA 96 h prior to the staining. Nuclei were counterstained with DAPI. Scale bar = 25 μm. **e** Immunostaining of Bex1 at different cell densities in MDCK cells. At a low cell density, Bex1 showed localization to nucleoli which were stained with FBL antibody. At a high cell density, Bex1 showed a condensed pattern which colocalized to α-tubulin. Nuclei were stained with DAPI. Scale bar = 25 μm. **f** The XY view and the corresponding reconstructed *z*-axis view of MDCK cells at the high cell density. The immunostaining showed localization of BEX1 to the apical side of cells where tubulin was enriched. Scale bar = 25 μm

**Fig. 4:**
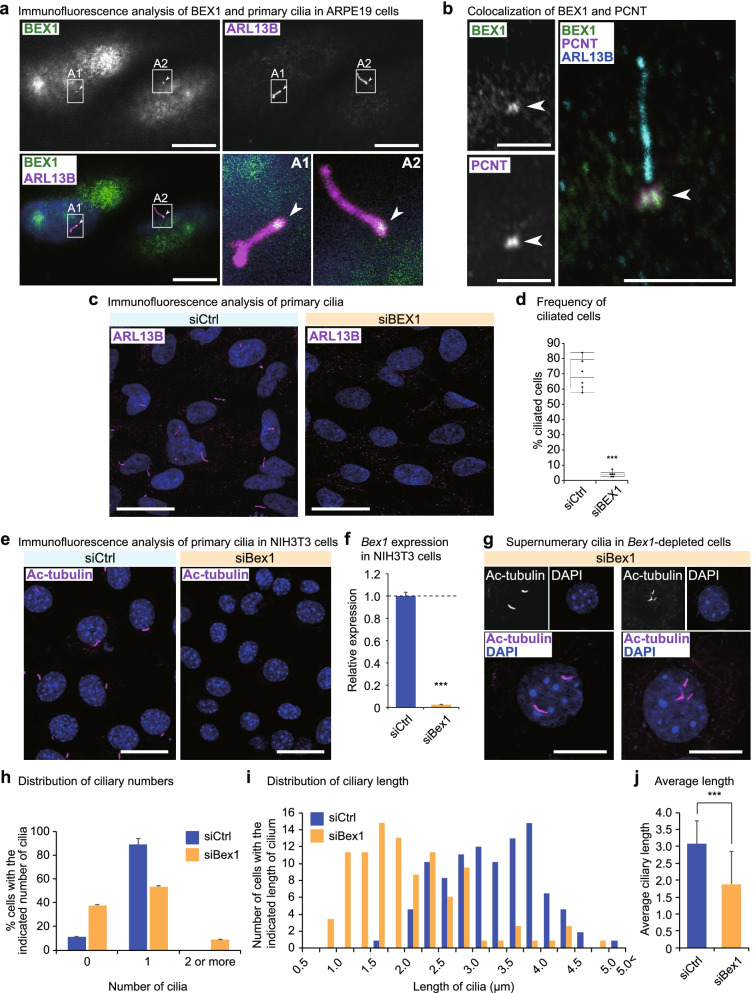
Bex1 localizes to the ciliary base and promotes ciliogenesis. **a** Immunofluorescence analysis of BEX1 and primary cilia in human ARPE19 cells. The primary cilia were stained with the ARL13B antibody. Bex1 localized to the base of primary cilia. Nuclei were stained with DAPI. Scale bar = 10 μm. **b** Immunofluorescence analysis of BEX1, PCNT and ARL13B in human ARPE19 cells. BEX1 and PCNT colocalize at the ciliary base. Scale bar = 5 μm. **c** Immunofluorescence analysis for the primary cilia in ARPE19 cells transfected with control siRNA or *BEX1* siRNA. Cilia formation was abrogated by the *BEX1* depletion. Nuclei were stained with DAPI. Scale bar = 25 μm. **d** Frequency of ciliated cells after transfection with control siRNA or *BEX1* siRNA. The number of ciliated cells was counted in the experiment (**c**). **e** Immunofluorescence analysis for the primary cilia in mouse NIH3T3 cells transfected with control siRNA or *Bex1* siRNA. Primary cilia were visualized with the acetylated tubulin (Ac-tubulin) antibody. Nuclei were stained with DAPI. Scale bar = 25 μm. **f** qPCR analysis for the expression of *Bex1* in NIH3T3 cells transfected with control siRNA or *Bex1* siRNA. Data were normalized to *Tubb5*. **g** Supernumerary cilia observed in *Bex1*-depleted cells. The primary cilia were stained with antibodies against Ac-tubulin and IFT88. Nuclei were stained with DAPI. Scale bar = 10 μm. **h** Distribution of the number of primary cilia in a cell after transfection with control siRNA or *Bex1* siRNA. A total of 9.1% of *Bex1*-depleted cells exhibited supernumerary cilia (denoted as “2 or more”). **i** Length (μm) distribution of the cilia in NIH3T3 cells transfected with control siRNA or *Bex1* siRNA. **j** Average length (μm) of the primary cilia in NIH3T3 cells after transfection of control siRNA or *Bex1* siRNA. ∗∗∗ *p* < 0.001; Student’s *t* test. The data are presented as the means ± standard deviations

The physical implication that Bex1 functions as an IDP led us to next examine the biological behaviours of Bex1. We first analysed the subcellular localization of BEX1 in ARPE19 cells. BEX1 showed cell density-dependent subcellular localization. At a low cell density in which at least one side of cells was unoccupied and did not contact other cells, BEX1 localized to nucleoli (Fig. 3a). The depletion of *BEX1* by siRNA transfection erased the nucleolar signal, supporting the specificity of the nucleolar signal to BEX1 (Additional file 1: Fig. S3a). The cytoplasmic signals were not suppressed by *BEX1* knockdown and thus considered to be nonspecific (Additional file 1: Fig. S3a). At a high cell density in which cells contacted other cells on every side, BEX1 translocated to the apical surface of cells, where Bex1 was associated with tubulin fibres (Fig. 3a,b). On the apical side of the cells, Bex1 also showed colocalization with primary cilia (Fig. 3a, “High density”, insets), as shown in subsequent experiments (Fig. 4). At a high cell density, ARPE19 cells form a columnar epithelial cell shape and develop apicobasal polarity. The columnar epithelial cells develop a dense microtubule network on the apical side of the cells, where BEX1 is localized (Fig. 3b). The knockdown of *BEX1* led to defective formation of the apical microtubule network and flattening of cell layers (Fig. 3b). Moreover, cells depleted of *BEX1* exhibited stratification, indicating that the cells lost their epithelial properties (Fig. 3c). The loss of epithelial properties was further confirmed by examining cell-cell junction proteins, and it was found that intercellular junctions formed by α-catenin and β-catenin were lost in *BEX1*-depleted cells (Fig. 3d). Cell density-dependent localization was also examined in Madin-Darby canine kidney (MDCK) cells, which form a well-polarized epithelial structure. Consistent with the findings in ARPE19 cells, Bex1 localized to nucleoli at a low cell density and to the apical microtubule network at a high cell density (Fig. 3e). Bex1 localized to the apical side of cells and showed colocalization with the microtubule network (Fig. 3f). These findings suggest a role of Bex1 in the formation of the apical microtubule network and the maintenance of apicobasal polarity.

We further investigated the biological relevance of the nucleolar localization of BEX1 at a low cell density. Nucleoli show an internal structure (Additional file 1: Fig. S3b, [38]) that can be delineated by staining with fibrillarin (FBL), which localizes to the dense fibrillar component (DFC [39];), and Nucleolin, which localizes to the granular component (GC [40];). BEX1 immunostaining revealed clearer overlap with FBL than with Nucleolin, suggesting that BEX1 localizes to the DFC (Additional file 1: Fig. S3). We next investigated the potential role of BEX1 in the DFC of the nucleoli. The depletion of *BEX1* caused perturbation of the FBL staining pattern, suggesting the occurrence of DFC deformation (Additional file 1: Fig. S3d). The sphericity of the FBL staining patterns was significantly reduced by *BEX1* depletion, suggesting that BEX1 plays a role in maintaining the morphology of the DFC (Additional file 1: Fig. S3e,f).

### BEX1 localizes to the ciliary basal body and is indispensable for ciliogenesis

We further analysed the apical localization of BEX1 in ARPE19 cells. ARPE19 cells develop primary cilia at a high cell density. We found that BEX1 localized to the base of the primary cilia (Fig. 4a). Further analysis according to the components of the primary cilia revealed that BEX1 colocalized with PCNT, a component of the pericentriolar matrix (Fig. 4b). *BEX1* knockdown abrogated the formation of the primary cilia, demonstrating the indispensable role of BEX1 in ciliogenesis (Fig. 4c,d and Additional file 1: S4a-c). We also investigate whether the cilia disruption was caused by loss of cell polarity. TGF-β treatment induces epithelial mesenchymal transition of ARPE19 cells and provokes loss of apicobasal polarity [41–43]. The cilia formation was not appreciably affected by the loss of apicobasal polarity in ARPE19 cells, indicating the ciliary loss observed in *BEX1*-depleted cells was not due to the loss of cell polarity (Additional file 1: Fig. S4d).

**Fig. 5:**
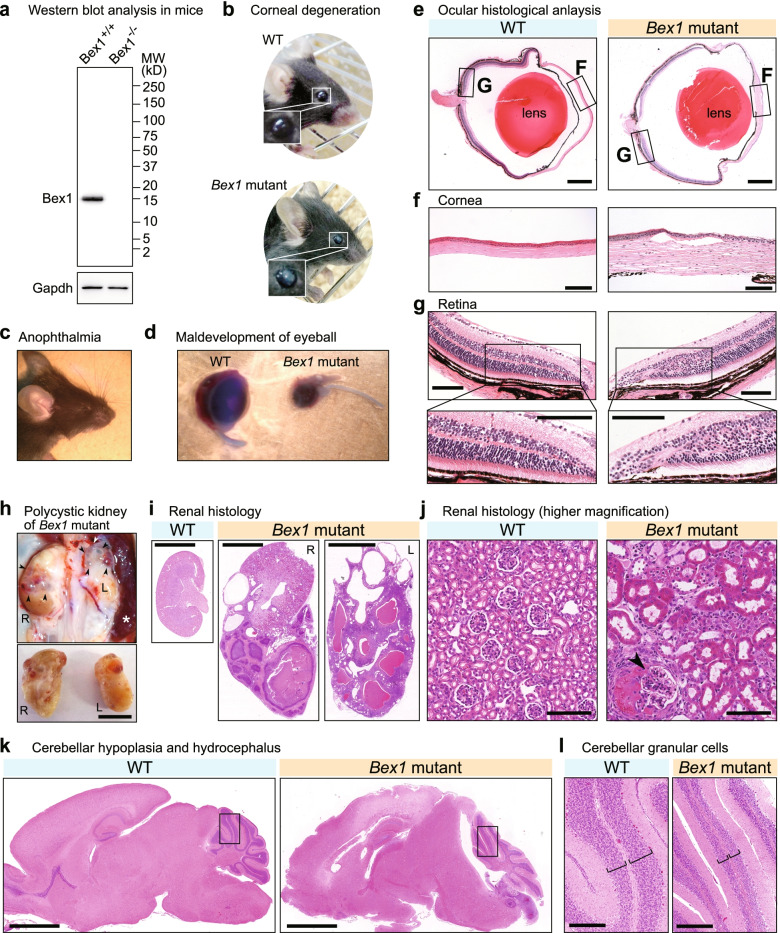
*Bex1* mutant mice exhibit ciliopathy phenotypes. **a** Western blot analysis for Bex1 in a Bex1 knockout and control wild type (WT) mice. The brain tissue lysates were analysed for Bex1 protein expression. Gapdh was used as loading control. **b** Ocular opacity caused by corneal degeneration in a *Bex1* mutant mouse. **c** Anophthalmia exhibited by a *Bex1* mutant mouse. **d** Maldevelopment of the eyeball caused by the *Bex1* mutation. The maldeveloped eyeball (right) was embedded in the connective tissue. An eyeball of a WT littermate is shown for comparison (left). **e** Ocular histological analysis of WT and *Bex1* mutant mice. Haematoxylin and eosin (HE) staining of the eyeballs is shown. Scale bar = 500 μm. **f** HE histological analysis of cornea showing abnormal stratification of corneal epithelium in *Bex1* mutant mice. Scale bar = 100 μm. **g** Retinal histological analysis showing the disorganized formation of retinal layers and malformation of retinal pigment epithelium (RPE) in *Bex1* mutant mice. Scale bar = 100 μm. **h** Polycystic kidney diseases caused by *Bex1* mutation. The upper panel shows kidneys (R and L) and spleen (*). Multiple cysts were observed in the kidneys of the *Bex1* mutant mouse. The arrowheads indicate cysts. The lower panel shows the appearance of the dissected kidneys (R and L). Scale bar = 1 cm. **i** HE staining of the WT and the *Bex1* mutant kidneys. Bilateral multiple cysts were observed. Scale bar = 4 mm. **j** Histological analysis at higher magnification shows dilatation of renal tubules and involuted glomerulus (arrowhead) in the *Bex1* mutant mouse. Scale bar = 100 μm. **k** Dysplasia of the cerebellar vermis accompanied by hydrocephalus with fourth ventricular enlargement in the *Bex1* mutant mouse. Scale bar = 2 mm. **l** Magnification of the squared areas in (**k**). The brackets indicate the hypoplasia of cerebellar granule cell layers in the *Bex1* mutant mouse. Scale bar = 200 μm

The primary cilium is not restricted to polarized epithelial cells and is also found in NIH3T3 cells, a type of mesenchymal cells (Fig. 4e). *Bex1* knockdown in NIH3T3 cells (Fig. 4f) compromised the formation of primary cilia (Fig. 4e). We noted that a small number of cells (9.1%) exhibited supernumerary cilia (Fig. 4g,h). The molecular aetiology of supernumerary cilia was subsequently investigated (Fig. 5). If cilia were detected at all, the length of the primary cilia was significantly shortened, indicating that ciliogenesis was compromised by *Bex1* depletion (Fig. 4i,j). Overexpression of BEX1 did not affect ciliogenesis and PCNT localization (Additional file 1: Fig. S4e, f). Thus, BEX1 protein localization at the base of the primary cilium is indispensable for proper ciliogenesis.

### *BEX1* depletion from the midbody provokes cytokinesis failure, underlying the formation of supernumerary cilia

The colocalization of BEX1 with apical microtubules and primary cilia led us to investigate the potential localization of BEX1 to the other tubulin-enriched subcellular apparatus. BEX1 immunostaining revealed the accumulation of BEX1 in the midbody, where tubulin forms dense microtubules in dividing ARPE19 cells (Additional file 1: Fig. S5a). *BEX1* knockdown hampered the formation of midbodies with typical tubulin accumulation (Additional file 1: Fig. S5b), resulting in the elongation of the cells as a result of compromised abscission in ARPE19 cells (Additional file 1: Fig. S5c).

Additionally, in NIH3T3 cells, we found that Bex1 localized to the midbody (Additional file 1: Fig. S5d). *Bex1* depletion led to abnormal nuclei with increased foci of PCNT, an essential component of the centrosome, suggesting that the failure of cell division led to the supernumerary centrosomes (Additional file 1: Fig. S5e,f). *BEX1* depletion caused the increased PCNT foci also in ARPE19 cells (Additional file 1: Fig. S5g). The centrosome functions as the basal body in the context of ciliogenesis. We actually observed that the increased number of centrosomes contributed to the formation of supernumerary cilia in *Bex1*-depleted NIH3T3 cells (Additional file 1: Fig. S5h,i).

Compared to the results obtained in ARPE19 cells, in which *BEX1* depletion abrogated ciliogenesis nearly completely, NIH3T3 cells exhibited remaining cilia with aberrant lengths and numbers (Fig. 4g-j), implying a compensatory mechanism for Bex1-dependent ciliogenesis. This finding prompted us to investigate the potential mechanism that compensated for cilium formation in the absence of Bex1 expression in NIH3T3 cells. Bex2 shared the highest structural similarity with Bex1 among the members of the Bex family and was expressed in NIH3T3 cells (Additional file 1: Fig. S6a). *BEX2* expression was low in ARPE19 cells (Additional file 1: Fig. S6b). Although *Bex2* depletion showed minor effects on ciliogenesis (Additional file 1: Fig. S6), the simultaneous depletion of *Bex1* and *Bex2* resulted in the complete suppression of supernumerary cilia, suggesting that Bex2 plays a compensatory role in ciliogenesis in the absence of Bex1 (Additional file 1: Fig. S6c,d). Thus, the increase in centrosomes due to *Bex1* depletion underlies the formation of supernumerary cilia, which is mediated by the compensatory role of *Bex2*.

### *Bex1* mutant mice exhibit ciliopathy phenotypes

We next sought to determine a role of Bex1 in vivo. We established *Bex1* mutant mice via CRISPR-Cas9 genome editing [44–46], Additional file 1: Fig. S7a). We utilized a strain with a frameshift mutation in *Bex1* (Additional file 1: Fig. S7b) in which we confirmed that no mutation occurred in *Bex2* (Additional file 1: Fig. S7c). The loss of the Bex1 protein was confirmed using a newly developed antibody that is responsive to mouse Bex1 (Fig. 5a). *Bex1*-null male^−/Y^ and female^−/−^ mice were viable and fertile.

Female mutant mice showed lateral or bilateral ocular turbidity more frequently than male mice (Fig. 5b and Additional file 1: Fig. S7d), indicating that *Bex1* shows sex-dependent biological relevance. The female mutants also showed a more severe phenotype causing maldevelopment of the eyeball manifesting as anophthalmia (the absence of an eye, Fig. 5c,d). Histological analyses of the eyeballs of WT and *Bex1* mutant mice revealed thickening of the corneal epithelium (Fig. 5e,f) and disoriented formation of retinal epithelial layers (Fig. 5g), in line with the in vitro findings in ARPE19 cells, in which *BEX1* knockdown led to a loss of apicobasal polarity and stratification of cells (Fig. 3c).

For the determination of the phenotypes, we focused on female homozygous mutant mice. The *Bex1* mutant mice also presented abdominal swelling that was caused by enlarged kidneys with multiple cysts (Fig. 5h). Renal histological analysis revealed large cystic lesions (Fig. 5i) and dilatation of the renal tubules, accompanied by glomerular involution, confirming that the mice were affected by polycystic kidney disease (Fig. 5j). Moreover, *Bex1* mutant mice exhibited an increased head circumference due to hydrocephalus accompanied by hypoplasia of the cerebellar vermis (Fig. 5k). The cerebellar vermis was primarily affected, although secondary hydrocephalus caused deformation of the entire cerebellum. The histological analysis of the cerebellum revealed thinning of the cerebellar granule cell layers (Fig. 5l). The motile cilia of the airway epithelium were not affected by *Bex1* mutation, confirming the physiological relevance of *Bex1* in primary cilia, not motile cilia (Additional file 1: Fig. S7e). Appreciable defects were not found in other tissues such as skin, heart, liver and gut. We did not observe worsening of phenotypes in aged animals. This constellation of symptoms (ocular dysgenesis, polycystic kidney disease and cerebellar hypoplasia) was in accord with ciliopathy phenotypes and suggested that the defects were due to ciliary dysfunction [47–49].

**Fig. 6:**
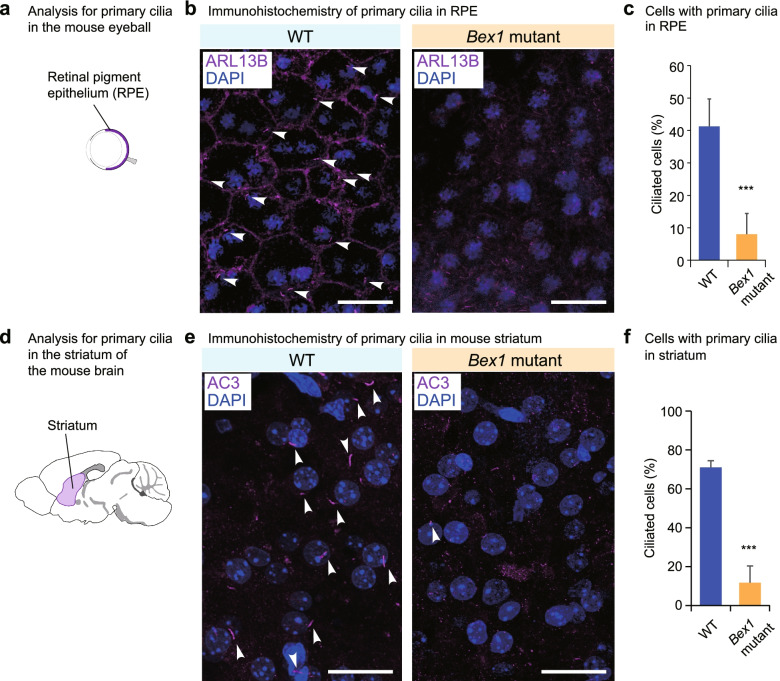
*Bex1* promotes ciliogenesis in mice. **a** Schematic representation of the retinal pigment epithelium (RPE) in which primary cilia were analysed. **b** Immunohistochemistry for the primary cilia stained with ARL13B antibody in the RPE of WT and *Bex1* mutant mice at P8. Nuclei were stained with DAPI. Scale bar = 10 μm. **c** Frequency of cells with a primary cilium in the RPE of WT and *Bex1* mutant mice. **d** Schematic representation of the striatum in the mouse brain in which primary cilia were analysed. **e** Immunohistochemistry for the primary cilia stained with Adenylate Cyclase 3 (AC3) antibody in the striatum of WT and *Bex1* mutant mice at P13. Nuclei were counterstained with DAPI. Scale bar = 25 μm. **f** Frequency of cells with a primary cilium in the striatum of WT and *Bex1* mutant mice. ∗∗∗ *p* < 0.001; Student’s *t* test. The data are presented as the means ± standard deviations

We next examined the influence of *Bex1* mutation on the formation of primary cilia in *Bex1* mutant mice. Here, we investigated the retinal pigment epithelium (RPE) of the eyeball (Fig. 6a) and the striatum of the brain (Fig. 6d) as tissues rich in primary cilia. The primary cilia of RPE cells are indispensable for the proper maturation and functions of the cells [50–52]. Immunostaining of cilia with an ARL13B antibody revealed significantly fewer primary cilia in the retinal pigment epithelial cells of *Bex1* mutant mice than in those of WT mice (Fig. 6b,c). Consistent with the findings in vitro, supernumerary cilia were observed in RPE of KO mice (Additional file 1: Fig. S7f). Additionally, in the striatum (Fig. 6d), significantly fewer primary cilia were found in *Bex1* mutant mice than in WT mice (Fig. 6e,f). These findings indicate that *Bex1* is indispensable for ciliogenesis in mice.

### The Bex1 condensate induces tubulin polymerization

**Fig. 7:**
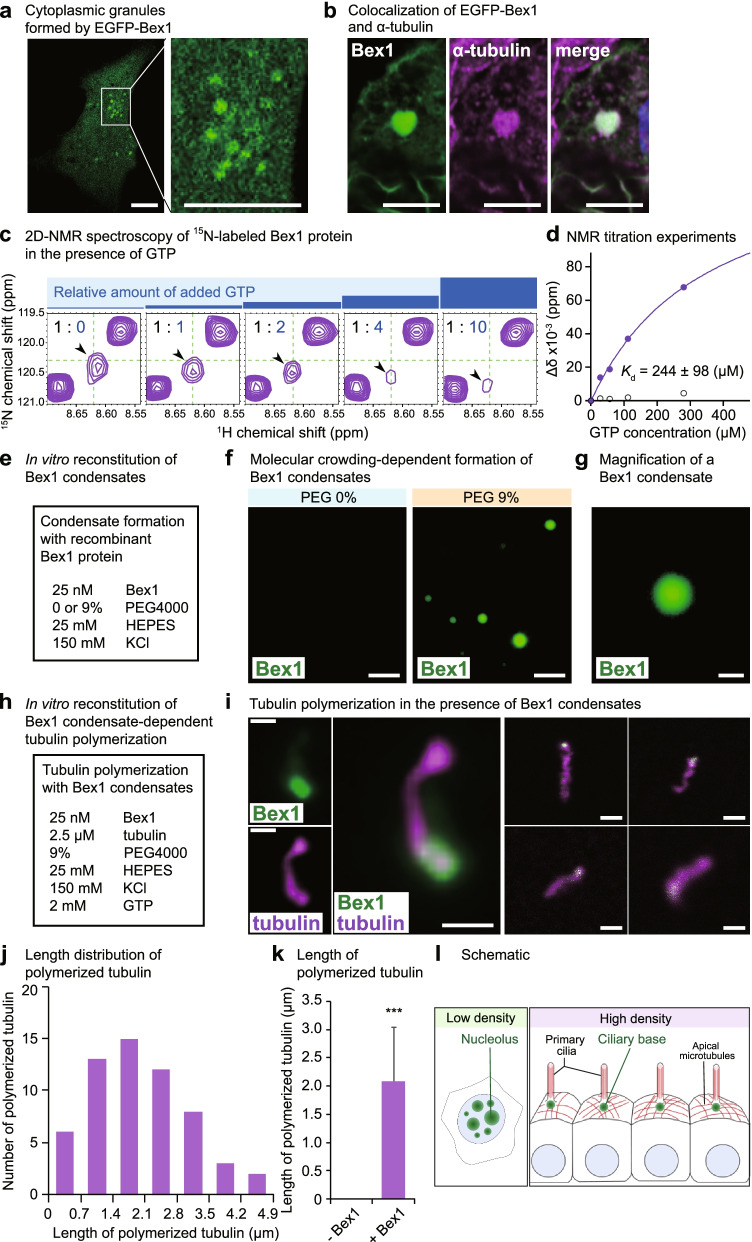
Bex1 reconstitutes tubulin polymerization. **a** Image derived from unfixed cells expressing EGFP-Bex1. EGFP-Bex1 forms cytoplasmic granules. Scale bar = 5 μm. **b** Immunostaining for α-tubulin in the cells expressing EGFP-Bex1. The EGFP-Bex1 granules accumulate α-tubulin. Scale bar = 5 μm. **c** The 2D-NMR spectroscopy with ^15^N-labelled recombinant Bex1 protein in the presence of indicated molar ratios of Bex1 to guanosine triphosphate (GTP). The ^1^H-^15^N HSQC spectra were measured at pH 7.3 and 25°C. The increased GTP provoked the peak shifts (indicated by arrowheads) in a dose-dependent manner. **d** Calculation of the dissociation constant (*K*_d_) for Bex1 and GTP. The distance of the peak shifts (Δδ) obtained in (C) was plotted as the filled dots to calculate the dissociation constant of Bex1 and GTP. For comparison, the plots from the upper right peak that did not shift were shown as the open dots. **e** Concentrations of reagents for the analysis of the Bex1 condensate formation. **f** Formation of Bex1 condensates in the presence or absence of PEG4000. Recombinant Bex1 protein was labelled with DyLight fluorescent dye. Scale bar = 5 μm. **g** The magnified view of the Bex1 condensate. Scale bar = 1 μm. **h** Concentrations of reagents for the analysis of tubulin polymerization in the presence of the Bex1 condensates. **i** Representative images of polymerized tubulin (magenta) provoked by the presence of the Bex1 condensates (green). Tubulin protein was labelled with Alexa fluorescent dye. Scale bar = 1 μm. **j** Histogram of the length (μm) of the polymerized tubulin in the presence of the Bex1 condensates. **k** Length (μm) of the polymerized tubulin. Without recombinant Bex1 protein, no tubulin polymerization was observed. **l** Schematic representation of the cell density-dependent role of BEX1 in regulating cellular homeostasis. At a low cell density, BEX1 localizes to nucleoli, where BEX1 contributes to the formation of DFCs. At a high cell density, BEX1 localizes to the apical side of cells and contributes to the formation of apical microtubules and primary cilia. ∗∗∗ *p* < 0.001; Student’s *t* test. The data are presented as the means ± standard deviations

To gain insight into how Bex1, an IDP, contributes to the formation of primary cilia, we analysed the functional property of Bex1 protein. We transfected *EGFP*-fused *Bex1* into ARPE19 cells and observed the cells under unfixed conditions. Fluorescent microscopy revealed that EGFP-Bex1 protein formed cytoplasmic granules (Fig. 7a). The Bex1 granules colocalized with tubulin, implying that the Bex1 granules concentrate tubulin (Fig. 7b). Fluorescent recovery after photo-bleaching (FRAP) analysis with the Bex1 granules revealed rapid recovery of fluorescence suggesting the dynamic exchanges of Bex1 molecules (Additional file 1: Fig. S8a,b). Further, to investigate the potential role of Bex1 in facilitating the tubulin polymerization, we investigated the interaction of Bex1 with GTP, a crucial substrate that tubulin utilize to polymerize. To examine the binding capacity of Bex1 with GTP, we performed 2D-NMR analysis in the presence of GTP. Upon GTP addition, Bex1 underwent significant changes in its chemical shift, indicating that Bex1 is bound to GTP (Fig. 7c and Additional file 1: Fig. S8c). The peak shift of Bex1 was GTP concentration-dependent with the dissociation constant (*K*_d_) equal to 244 ± 98 μM (Fig. 7d). We also tested the binding of Bex1 to another nucleotide, ATP. The 2D-NMR analysis showed that the addition of ATP changed the spectrum of ^15^N-labelled Bex1, indicating that Bex1 also interacted with ATP. However, unlike GTP, ATP did not show a concentration-dependent peak shift, rather, the addition of 1 and 2 equivalents of ATP decreased the peak intensity of Bex1 whereas 4 and 10 equivalents increased, indicating the chemical exchange between Bex1 and ATP was slower than that between Bex1 and GTP (Additional file 1: Fig. S8d).

We utilized an assay system in which extracted tubulin underwent polymerization under reconstituted experimental conditions. The extracted tubulin did not undergo polymerization without an appropriate supply of tubulin and GTP. The purification method that we established enabled us to investigate the role of the recombinant Bex1 protein in its native state. The recombinant Bex1 protein was purified and covalently labelled with DyLight fluorescent dye. After dialysis to remove unreacted DyLight molecules, the labelled Bex1 protein was incubated in the presence or absence of polyethylene glycol (PEG4000, Fig. 7e). In the presence, but not in the absence of PEG, the Bex1 protein formed molecular condensates (Fig. 7f, g). The requirement for PEG indicated the necessity of the macromolecular crowding effect for Bex1 condensate formation. The FRAP analysis revealed recovery of the fluorescence suggesting the presence of exchanges of Bex1 protein in the condensates (Additional file 1: Fig. S8e). Next, to determine the role of the Bex1 protein in ciliogenesis, we assessed whether the presence of Bex1 condensates was sufficient for the induction of tubulin polymerization. We incubated Bex1 condensates with tubulin protein fluorescently labelled with Alexa 488 (Fig. 7h). In the presence of Bex1 condensates, the tubulin protein underwent a polymerization reaction, indicating that the Bex1 condensate provided the appropriate the microenvironment for tubulin polymerization (Fig. 7i, j). In the absence of the Bex1 condensate, tubulin monomers did not undergo polymerization (Fig. 7k). Thus, Bex1 forms molecular condensates when a molecular crowding effect exists. The Bex1 condensates were sufficient to provoke tubulin polymerization under reconstituted conditions. The tubulin polymerization is pivotal machinery for the formation of the primary cilia, thus linking the physiological role of Bex1 to the ciliogenesis.

We characterized Bex1 as a juvenile-expressed IDP that showed cell density-dependent localization to nucleoli and the apical side of cells, where Bex1 localizes to the basal body (Fig. 7l). Bex1 promotes the formation of primary cilia in both cells and mice, and Bex1 mutations cause ciliopathy phenotypes in mice. The Bex1 protein is capable of forming condensates that facilitate tubulin polymerization.

## Discussion

In this paper, we have described a role of Bex1 involving cell density-dependent localization to either nucleoli or the apical side of cells. Nucleolar Bex1 was found to be located in DFCs and to be indispensable for the maintenance of DFC morphology. The colocalization of Bex1 with apical microtubules was clearly observed in confluent and polarized epithelial cells. In ARPE19 cells, the localization of BEX1 was specifically found at the base of the primary cilia. Immunostaining studies revealed the colocalization of BEX1 with PCNT, which is a fundamental component of the basal body, indicating that BEX1 functions for the formation of primary cilia.

Knockdown experiments in cultured cells demonstrated that Bex1 promotes proper ciliogenesis. Mutant mouse analyses revealed that Bex1 mutation compromised the primary cilia in the RPE and the striatum, but not motile cilia in the airway epithelium, indicating an essential role specific to primary cilia. Consistent with this, *Bex1* mutant mice displayed ciliopathy phenotypes. In the retina, the *Bex1* mutation resulted in disoriented epithelial structures (Fig. 5G), which was in line with the in vitro findings in *BEX1*-depleted ARPE19 cells (Fig. 3C). Cerebellar vermis hypoplasia was previously explained by the impaired growth capacity of cerebellar granule cells due to ciliary defects [53, 54].

*Bex1* mutant mice showed sex differences, with female mutant mice (X^mutant^/X^mutant^) presenting more severe phenotypes than male mice (X^mutant/^Y). We therefore performed more detailed phenotypic analyses in female mice. The sex-dependent variability of the phenotypes may explain why previous research on *Bex1* mutant mice did not identify ciliopathy phenotypes. Koo et al. specifically analysed male mice [5], and Accornero et al. performed cardiac pressure overload experiments in male mice [6]. The mechanism underlying this sex difference remains to be demonstrated. Further comparisons between the sexes will advance our understanding of the sex-dependent biological function of Bex1. Karp et al. performed an extensive analysis of sex differences in 14,250 WT and 40,192 mutant mice from 2,186 knockout lines of the International Mouse Phenotyping Consortium (IMPC) and showed that 56.6% of the datasets included sex differences, among which 26.6% of the phenotypes were stronger in females. Notably, half of the datasets showed stronger “eye” phenotypes in females [55]. The molecular mechanisms underlying these sex differences are still elusive, although they hold potential from biological and clinical viewpoints.

BEX1 colocalized to apical microtubules in ARPE19 and MDCK cells. *BEX1* depletion abrogated apical microtubules and induced the loss of epithelial properties, which manifested in cell scattering and abnormal cell stratification. Microtubule cytoskeleton in cytosol was not affected appreciably by *BEX1* knockdown. The frequent association of BEX1 with tubulin-enriched cell structures led us to find the accumulation of BEX1 in the midbody. *BEX1* depletion prevented cytokinesis and caused abscission failure in ARPE19 cells and increased the number of centrosomes in NIH3T3 cells. The supernumerary centrosomes underlie the formation of supernumerary cilia, which is mediated by the compensatory role of Bex2. Bex1 depletion did not cause loss of PCNT signals, suggesting centrosomes are formed in these cells. Because Bex1 binds GTP and promote microtubule polymerization in the reconstituted system, we consider that Bex1 contributes to the maturation of the centrosomes by providing an efficient reaction field that enriches GTP for promoting the microtubule polymerization. Different dependency on *Bex1* was seen in ARPE19 and NIH3T3 cells. Whereas *BEX1* depletion led to nearly complete loss of cilia in ARPE19 cells, *Bex1* knockdown provoked supernumerary cilia in some (9.1%) of NIH3T3 cells. The relevance of this finding in vivo might be the tissue selectivity of the manifestation of *Bex1* mutant mice. *Bex1* mutation led to the pathogenesis in the retina, cerebellum and kidney. The different dependency on *Bex1* might underly the tissue-specific manifestation observed in mice.

Bex1 localizes to nucleoli at low cell density. Bex1 does not possess a nuclear localization signal (NLS) or a nucleolar localization signal (NoLS). The biological relevance of the nucleolar localization of Bex1 at low cell density has not been elucidated, but we speculate that Bex1 might interact with GTP, substrates for RNA synthesis, in nucleoli. Compared to the cells at high density, cells at low density are dividing more rigorously and tend to develop prominent nucleoli. Bex1 might contribute to the RNA synthetic function in nucleoli, which will be investigated in a future study.

To reveal the molecular function of Bex1, we adopted the unbiased approach of starting the study by analysing its amino acid sequence. This clarified the characteristics of the Bex1 protein, which did not contain any annotated domains, indicating a new principle of action. The detailed sequence analysis identified an IDR in the Bex1 amino acid sequence. For the chemical analyses, we carefully avoided any denaturing steps to allow us to investigate the native state of the Bex1 protein in the assays. The preceding work by Fernandez et al. involved denaturing steps including boiling and measurements under acidic (pH 5.1) conditions [34], thus hampering the investigation of the physiological properties of Bex1. Measurements performed at pH 7.3 and at different temperatures, including 37°C, verified the absence of stable secondary structures in Bex1, demonstrating that Bex1 is an IDP. Consistent with this notion, the recombinant Bex1 protein exhibited the capacity to undergo condensate formation in the presence of a molecular crowding agent.

We also investigated the interaction of Bex1 with GTP, an essential substrate for tubulin to catalyse in the polymerization reaction. The 2D-NMR spectroscopy revealed the dose-dependent peak shifts of Bex1 in the presence of GTP. The 2D-NMR analyses revealed that the chemical exchange between Bex1 and ATP was slower than that between Bex1 and GTP, implying the preferential binding of Bex1 to GTP. The colocalization of EGFP-Bex1 with α-tubulin implied that Bex1 might interact with α-tubulin, but we thus far have not detected the direct interaction of Bex1 with tubulin. On the basis of the advantages of nondenatured recombinant Bex1 purification method, the sufficiency of the Bex1 protein for inducing tubulin polymerization was assessed in tubulin polymerization assays. Woodruff et al. induced robust microtubule polymerization by incubating SPD-5 condensates with TPXL-1 and ZYG-9 [29]. The microtubule polymerized in the presence of Bex1 condensates appear wiggled and thinner compared to the microtubule generated in the presence of SPD-5/TPXL-1/ZYG-9. Because SPD-5 and ZYG-9 induced the microtubule polymerization more infrequently, addition of another factor to Bex1 condensates might facilitate the microtubule polymerization. Liquid-like behaviours of ciliary bases have previously been suggested in motile cilia [56] but not yet in primary cilia. Our findings implied that Bex1 forms subcellular compartments at tubulin-enriched sites and ciliary bases, where Bex1 associates with the microtubule polymerization substrates.

We focused on juvenile-expressed genes with the aim of revealing a mechanism that differentiates juveniles from adults. The study led us to identify an indispensable role of *Bex1*, a JAG, in the formation of primary cilia. In the retina, the presence of primary cilia decreases as postnatal maturation continues, implying a role of Bex1 in maintaining retinal primary cilia in a specific life stage [50, 57]. Elucidating the purpose for which primary cilia are formed only in juveniles is a goal of future work, but it is possible that cilia may sense external cues necessary to optimize tissue maturation.

*Bex1* mutant mice exhibited ciliopathy phenotypes, manifested in the cornea, retina, kidney and cerebellum, forming a constellation of symptoms reminiscent of Joubert syndrome [47, 48]. More than 35 genes are known to cause Joubert syndrome when mutated [58]. No *Bex1* mutation has yet been reported to cause human diseases, but a future genetic investigation may reveal a role of the *Bex1* gene in disease causation.

Different types of cilia contribute to organ development. As far as we observed, *Bex1* mutation compromises the primary cilia in the retina and striatum. The cilia of these tissues are known to be nonmotile without a central pair of microtubules (noted as “9+0”,[59]). In the airway epithelium where motile 9+2 cilia were formed, the motile cilia were not appreciably affected by *Bex1* mutation. Left-right asymmetry is introduced by motile 9+0 cilia that produce the nodal flow. As far as we have investigated, the *Bex1* mutant mice did not exhibit randomization of left–right asymmetry (situs inversus), suggesting *Bex1* was not essential for this context. These findings indicate the different types of cilia were generated by varying machinery. Similarly, each human ciliopathy presents the variable constellation of symptoms, indicating the contribution of a particular cilia-related gene to the specific organs. Delineation of the molecular machinery underlying different types of cilia consists a future important task.

Primary cilia are functionally relevant in the retina, kidney and cerebellum. In the RPE, cilia have been reported to be necessary for the appropriate formation of retinal layers [51, 60, 61]. In the cornea, primary cilia control the proliferation of corneal epithelial [62] and endothelial [63] cells during corneal development. In the kidney, primary cilia of the renal tubular epithelium sense fluid flow and transduce mechanical signals to stimulate the cellular response of either proliferation or apoptosis [14]. Polycystic kidney disease or renal tubular malformation is another representative manifestation of ciliary defects [64]. Primary cilia were also investigated in the brains of *Bex1* mutant mice. Hypoplasia of the cerebellar vermis causing hydrocephalus was observed in *Bex1* mutant mice. The growth of cerebellar granule cells is known to be controlled by primary cilia [53, 54]. Our findings in *Bex1* mutant mice will contribute to further understanding the roles of primary cilia in tissue morphogenesis.

## Conclusions

In conclusion, we revealed an essential role for Bex1 in ciliogenesis both in cells and mice. Bex1 harbours the physicochemical property as an IDP and forms biomolecular condensates which are sufficient for the induction of tubulin polymerization. In cells, Bex1 localizes to the basal body thereby providing the reaction field for the macromolecular assembly. Bex1 exhibit binding capacity to GTP that is an essential substrate for tubulin polymerization. Loss of Bex1 causes the ciliopathy phenotypes in mice thus linking the Bex1’s molecular property to organogenesis. Our work focuses on the juvenile-specific molecular machinery and gives an insight into the basic principle how the mixture of molecules is built up into life.

## Methods

### Establishment of *Bex1* mutant mice

#### Reagents for genome editing

The *Bex1* mutant mouse strains were generated using CRISPR-Cas9 technology as described previously [46]. The gRNA was designed using CRISPRdirect (https://crispr.dbcls.jp/). The sequence of the gRNA is 5′-GTCGCAGGCGGTTCCGGGTTCGG-3′. The *Bex2* has the identical gRNA-targeting sequence. We confirmed *Bex2* was not mutated in all the strains we analysed. The gRNA sequence was cloned into the gRNA cloning vector (Addgene Plasmid ID #41824).

For gRNA synthesis, the T7 RNA polymerase recognition site was attached to the gRNA sequence via the polymerase chain reaction (PCR). The PCR products were purified and used as the template for in vitro RNA synthesis using the mMESSAGE mMACHINE T7 Transcription Kit (Life Technologies). The synthesized gRNA was purified using MEGAclear (Ambion). Recombinant Cas9 protein was purchased (GeneArt Platinum Cas9 Nuclease, Thermo Scientific, B25642).

#### Microinjection

Mouse zygotes were obtained via in vitro fertilization (IVF) of WT C57BL6/N gametes. gRNA (100 ng/ml) and Cas9 protein (30 ng/ml, TrueCut Cas9 Protein v2, Invitrogen) were mixed and microinjected into the pro-nuclei and cytoplasm of zygotes. The injected embryos were incubated at 37°C until they were transferred into pseudo-pregnant females at the two-cell stage.

#### Genotyping and breeding

Genomic DNA was extracted from the tail tips of pups, and the genomic sequence around the gRNA target site was PCR-amplified using the following primers: forward 5′-TGGCAAAAACTGTGCACCTA-3′ and reverse 5′-GGTCCCCATGTCATCTTCAG-3′. The PCR products were treated with ExoSAP-IT (Thermo Fisher Scientific) and sequenced with the forward primer. For the analyses of *Bex1* mutant mice, at least 4 different strains were analysed and gave rise to similar observations. In this paper, only the Δ7 strain was used for the analyses. The female at the F1 generation having confirmed mutation was first crossed to C57BL/6N male mice to refresh the Y chromosome. The F2 mutant male mice were then crossed with C57BL/6N female mice to refresh the X chromosome and mitochondrial genome for more than 8 generations. The Bex1 mutant allele of the strain harbouring 7-nucleotide deletion we mainly described in this paper was deposited at Mouse Genome Informatics with the allele number Bex1<em1Mori> MGI:6509427.

### Expression analysis of Bex family genes

The expression of Bex family genes was analysed utilizing the datasets we deposited with the accession number DRA007101 in the DDBJ Sequence Read Archive (DRA). Prenatal (embryonic day E18.5) and postnatal (postnatal day P1, P7, P14 and P56) gene expression in C57BL/6N mice (*n* = 3) were processed using Bio-Linux 8 [65] to examine the expression levels in FPKM values. Heatmaps were generated using MeV (http://mev.tm4.org).

### Cell culture

ARPE19 human retinal pigment epithelial cells were cultured in DMEM:F12 medium containing 10% foetal bovine serum (FBS) and penicillin/streptomycin. Neuro2a cells were cultured in Eagle's minimal essential medium supplemented with non-essential amino acids, 10% FBS and penicillin/streptomycin. NIH3T3 cells were cultured in DMEM containing 10% FBS and penicillin/streptomycin. In the knockdown experiments, Lipofectamine RNAiMAX (Invitrogen) was used to transfect the siRNA duplexes at 10 nM: human *BEX1* siRNA (Sigma-Aldrich, siRNA IDs: SASI_Hs01_00052058, SASI_Hs01_00052059, SASI_Hs01_00052061), mouse *Bex1* siRNA (Bioneer, AccuTarget siRNA ID1330657), mouse *Bex2* siRNA (Sigma-Aldrich, siRNA ID SASI_Mm01_00072434) and siRNA negative control (Applied Biosystems, AM4611). The knockdown efficiency was assessed 48 h after transfection. The cellular growth was assessed by counting the number of cells 72 h after transfection. The phase-contrast images of the cells were taken with an EVOS FL microscope (Thermo Fisher Scientific). 

### Gene expression analysis (qPCR)

The total RNA was extracted using TRIzol reagent (Thermo Fisher Scientific) from cultured cells according to the manufacturer’s instruction. The extracted RNA was quantified using a NanoDrop Lite Spectrophotometer (Thermo Fisher Scientific) and reverse transcribed using a High-Capacity RNA-to-cDNA Kit (Applied Biosystems) and a PCR Thermal Cycler Dice (Takara) according to the manufacturer’s instructions. qPCR was performed using a LightCycler 480 SYBR Green I Master Kit on a LightCycler 480 instrument (Roche) using the reverse-transcribed cDNA as a template. The specificity and quality of the qPCR amplification was assessed by performing a melting curve analysis. The data were normalized to human *TUBB* or mouse *Tubb5* as indicated in the Figure legends. The sequences of the primers are as follows: mouse *Bex1* 5′-GGAGCAGGTCTGAGAAGCAG-3′ and 5′-CACGCCTTGATCTTTGGACT-3′; mouse *Bex2* 5′-TGACTGGAAACCGAGAGTCC-3′ and 5′-CCTCCTTTTCCTGATGGTCA-3′; mouse *Tubb5* 5′-GATCGGTGCTAAGTTCTGGGA-3′ and 5′-AGGGACATACTTGCCACCTGT-3′; human *BEX1* 5′-AGAATCGGGAGAAGGAGGAG-3′ and 5′-TTTCTTGGTTGGCATTTTCC-3′; human *TUBB* 5′-TGGACTCTGTTCGCTCAGGT-3′ and 5′-TGCCTCCTTCCGTACCACAT-3′.

### Immunocytochemistry

ARPE19 cells, Neuro2a and NIH3T3 cells were fixed with 4% paraformaldehyde (PFA) for 10 min at room temperature (RT), permeabilized with 0.1% Triton X-100 for 2 min, blocked with 2% FBS at RT and incubated with antibody against Bex1 (ProteinTech, 12390-1-AP, 1:100), α-catenin (CST, #3240, 1:200), β-catenin (CST, #8480, 1:200), Vimentin (Santa Cruz, sc-6260, 1:100), Filamin (Thermo Scientific, #MS-1211, 1:40), Fibrillarin (FBL, 38F3, Novus, #NB300-269, 1:500), Nucleolin (CST, #87792S, 1:400), ARL13B (Abcam, ab1366481:100), acetylated tubulin (Sigma, T6793, 1:500), IFT88 (ProteinTech, 13967-1-AP, 1:100), PCNT (Abcam, ab4448, 1:500), PCNT-Alexa 594 (Novus, NB100-61071AF594, 1:200), LIN28B (CST, #4196S, 1:200), G3BP1 (Bethyl, A302-033A, 1:500) and tubulin (CST, #2148, 1:200) at 4°C overnight. After washing with PBS for 10 min 3 times, cells were incubated with appropriate secondary antibodies anti-rabbit-Alexa Fluor 488, anti-mouse-Alexa Fluor 594 or anti-mouse-Alexa Fluor 647 (1:1,000, Invitrogen) at RT for 1 h. Slides were mounted with Prolong Gold with DAPI (Invitrogen). Images were taken by confocal microscope TCS SP8 (Leica). For the analysis of the primary cilia, the frequency of ciliated cells and the number of cilia per cell were counted. Because a cell density or cell cycle status can influence ciliogenesis, the analysis of cilia was performed at similar cell density both for the normal and the *Bex1*-depleted conditions. The length of the primary cilia was measured with Leica Application Suite X software. The sphericity of nuclei was calculated with the images of Fibrillarin immunostaining, using the circularity plugin of the NIH ImageJ Fiji [66] using the formula: circularity = 4π(area/perimeter^2^).

### Western blot analysis with mouse tissues

For sampling mouse tissues, dissected tissues were crushed using a homogenizer (μT-12, Taitec) in chilled RIPA buffer containing 25 mM Tris-HCl (pH 7.6), 150 mM NaCl, 1% NP-40, 1% sodium deoxycholate and 0.1% SDS. The lysates were centrifuged at 20,400×*g* for 10 min to remove debris. Laemmli sample buffer was added to the lysates, followed by boiling at 95°C for 2 min. The protein samples (10 μg per lane) were separated by SDS-PAGE and transferred to a Hybond-P PVDF membrane (GE Healthcare). Because an antibody reactive to mouse Bex1 protein was not commercially available, we developed a new antibody reactive to mouse Bex1. Polyclonal antisera were generated by immunizing rabbits with the peptide [NH_2_-C+KKEEKEEKPQDTIR-COOH] derived from the Bex1-specific amino acid sequence. The N terminal cysteine [C+] of the peptide was added for the purpose of conjugating the peptide to carrier protein. As loading control, alpha-tubulin (1:4000, Cell Signaling Technology, #3873) and Gapdh (1:4000, Cell Signaling Technology, #5174) were used. Secondary antibodies conjugated with horseradish peroxidase (Thermo Fisher Scientific, anti-mouse 32430 and anti-rabbit 32460) were used at 1:1000 dilution. The immunoreactive bands were detected using Chemi-Lumi One L or Chemi-Lumi One Ultra (Nacalai Tesque, Kyoto, Japan).

### Mouse histological analysis

The ocular histology was analysed by the ophthalmologists. Following the euthanization of the mice, the eyes were enucleated and were fixed with 4% PFA at RT for 30 min. For haematoxylin and eosin (HE) staining, samples were paraffin-embedded and sectioned at 3 μm thickness. The images were taken by Nikon ECLIPSE 90i (Nikon). For immunohistochemistry, after removal of the optic nerve, cornea and lens, the retina was cut into quadrants, making angles at 90 degrees to the optic nerve head at the centre. The flat-mounted retina was analysed by immunohistochemistry as described below.

For the histological analysis in brains and kidneys, the mice were systemically fixed with 4% PFA followed by dissection of the organs. The dissected organs were post-fixed with 4% PFA for 3 h followed by sectioning at 3 μm thickness. The tissue sections were stained with HE. The images were taken by NanoZoomer S210 slide scanner (Hamamatsu) and analysed with NanoZoomer U12388-01 software (Hamamatsu). Histological diagnosis was made by the nephrologist.

For the immunohistochemistry with the retina and striatum, the flat-mounted retina or the PFA-fixed brain was permeabilized with 0.1% Triton X-100 for 2 min and blocked with 2% FBS at RT for 60 min. The tissues were incubated with the primary antibody against acetylated tubulin (Sigma, T6793, 1:500) at 4°C overnight. After washing with phosphate buffer saline (PBS), the tissues were incubated with the secondary antibody anti-mouse-Alexa Fluor 594 (1:1,000, Invitrogen). After washing with PBS, the tissues were mounted with Prolong Gold with DAPI (Invitrogen). Images were taken by confocal microscope TCS SP8 (Leica). For the analysis of the primary cilia, the frequency of ciliated cells was counted. The length of the primary cilia was measured with Leica Application Suite X software.

### Protein expression and purification

The expression plasmid for mouse *Bex1* were constructed with fusion proteins tagged with hexahistidine (His) at the N-terminus by inserting *Bex1* into the pETDuet-1 vector. The pETDuet-1-*Bex1* vector was introduced into the *E. coli* strain Rosetta-gami 2(DE3)pLysS. Transformed bacteria were grown in Luria broth medium containing ampicillin. Uniformly ^15^N-labelled proteins were grown in M9 minimal medium with 0.5 g/L ^15^NH_4_Cl. Bacterial cells were grown to an OD_600_ of ~ 0.6 at 37°C, and then isopropyl-β-D-thiogalactopyranoside (IPTG) was added to the medium. Cells were further incubated at 37°C for 5 h and were then harvested by centrifugation at 5000×*g* for 10 min at 4°C. The collected bacterial cells were suspended in the buffer containing 20 mM sodium phosphate (pH 8.0), 1 M NaCl, 0.2 mg/mL lysozyme, DNase I and 1 mM phenylmethanesulfonyl fluoride (PMSF) and were rotated for 1 h at 4°C. The cells were sonicated three cycles with intermittent pulses for 1 min (pulse of 0.7 s, interval of 0.3 s, output level of 3) using an ultrasonic disruptor equipped with a standard flat tip (Sonifier 450 Advance, Branson Ultrasonics, Connecticut). After removal of cell debris by centrifugation at 12,000×*g* for 45 min at 4°C, the cell extract was loaded onto a Ni-NTA resin (QIAGEN). The column was washed with a solution containing 20 mM sodium phosphate (pH 8.0), 300 mM NaCl and 20 mM imidazole, followed by the elution with the buffer containing 20 mM sodium phosphate (pH 8.0), 300 mM NaCl and 500 mM imidazole. The purity of the protein was analysed by SDS-PAGE. The purified protein was collected, dialyzed against suitable solution for each experiment and concentrated with a powder of absorbent gel (Spectra/Gel® Absorbent, Spectrum-Labs, Rancho Dominguez, CA, USA) at 4 °C. The concentration of Bex1 protein was calculated from the spectra measured by NanoDrop One (Thermo Scientific), using an absorption coefficient of 7000.

### CD spectroscopy

Circular dichroism CD spectra between 195 and 250 nm were collected on a J-805 spectropolarimeter (JASCO, Tokyo, Japan) at 10, 25, and 37 °C. We measured 200 μL solution of 5-10 uM BEX1 in 20 mM sodium phosphate buffer, 140 mM NaCl, pH7.3 in a quartz cuvette with a 1 mm light path length. The results were expressed as apparent ellipticity with the unit of ellipticity.

### Nuclear magnetic resonance (NMR) experiments

The two-dimensional ^1^H-^15^N HSQC spectrum was measured with a Bruker Avance III 900-MHz spectrometer equipped with a cryomagnetic probe. NMR experiments were performed at the protein concentration of 28 μM. The solvent conditions were 20 mM sodium phosphate buffer, 140 mM NaCl, 5% D_2_O, pH7.3. The chemical shift values were referenced to 4,4-dimethyl-4-silapentane-1-sulfonic acid (DSS).

In the NMR titration experiment with Bex1 and GTP, GTP was added to 28 μM Bex1 at concentrations of 28 μM, 56 μM, 112 μM and 280 μM, and each ^1^H-^15^N HSQC spectrum was measured for 2.5 h at 25 °C. Chemical shift perturbation (Δδ) was calculated by the following equation (1);

1 $$\Delta \updelta =\sqrt{{\left({\Delta \updelta}_{\mathrm{H}}\right)}^2+{\left(\Delta {\updelta}_{\mathrm{N}}/5\right)}^2}$$

where Δδ_H_ and Δδ_N_ are the chemical shift differences in the presence and absence of the GTP, respectively.

The concentration of GTP was plotted on the horizontal axis against Δδ on the vertical axis and fitted to obtain binding constant (*K*_d_) by the following eq. (2);

2 $${\Delta \updelta}_{\mathrm{H},\mathrm{N}}={\Delta \updelta}_{\mathrm{max}}\times \frac{\left(\left[\mathrm{Bex}1\right]+\left[\mathrm{GTP}\right]+{K}_{\mathrm{d}}\right)-\sqrt{{\left(\left[\mathrm{Bex}1\right]+\left[\mathrm{GTP}\right]+{K}_{\mathrm{d}}\right)}^2-4\left[\mathrm{Bex}1\right]\times \left[\mathrm{GTP}\right]\ }}{2\times \left[\mathrm{Bex}1\right]}$$

where Δδ_max_ is Δδ_H,N_ in case of moving to the maximum, and Δδ_max_ and *K*_d_ were calculated by fitting Eq. (2) using Igor application.

### Structural modelling

The tertiary structures of Bex family proteins were assessed by Phyre2 (http://www.sbg.bio.ic.ac.uk/phyre2/html/page.cgi?id=index). The property of Bex family proteins as intrinsically disordered proteins (IDPs) was assessed by PrDOS (http://prdos.hgc.jp/) and DISOPRED3 (http://bioinf.cs.ucl.ac.uk/psipred/).

### In vitro reconstitution assays

The Bex1 condensate formation was tested by the method described previously [32]. The recombinant Bex1 protein (10 μM) was labelled with DyLight 594 NHS ester according to the manufacturer’s instruction (ThermoFisher Scientific). To remove unbound dye, dialysis was performed with the dialysis buffer containing 25 mM HEPES and 150 mM KCl using BioDesignDialysis Tubing (molecular weight cut off = 8000 Da, BioDesign Inc. of New York). The labelled Bex1 protein (final concentration = 25 nM) was diluted in the buffer containing 25 mM HEPES, 150 mM KCl, 0.5 mM dithiothreitol (DTT) in the presence (9%) or absence (0%) of polyethylene glycol (PEG) 4000 and incubated for 5 min at 25°C. Images of the Bex1 condensates were taken by IX83 inverted microscope (Olympus). Pseudo-coloured images were generated to show Bex1 signals in green.

The potency of the Bex1 condensates to promote tubulin polymerization was assessed by incubating the Bex1 condensates with tubulin protein. For the tubulin, Alexa 488-labelled (catalogue number: 048805, PurSolutions, Tennessee) or unlabelled (catalogue number: 032005) tubulin was purchased and mixed at 1:4. The Bex1 condensates were incubated with the mixed tubulin at 2.5 μM in the buffer containing 25 mM HEPES,150 mM KCl, 0.5 mM DTT, 9% PEG and 2 mM GTP and incubated for 10 min at 25°C. Images of polymerized tubulin were obtained by IX83 inverted microscope (Olympus) and confocal microscope TCS SP8 (Leica). Pseudo-coloured images were generated to show Bex1 signals in green and tubulin signals in magenta.

### Fluorescent recovery after photo-bleaching (FRAP)

For the in vivo experiments, FRAP of EGFP-Bex1 in ARPE19 cells was performed on Fluoview FV3000 confocal microscope system (Olympus). Using a 60× oil immersion objective, a whole EGFP-Bex1 granule was bleached using a laser intensity of 20% at 488 nm. Recovery was recorded for every second for a total of 300 s after bleaching. Analysis of the recovery curves was carried out with cellSens software (Olympus).

For the in vitro experiments, FRAP was carried out with samples in glass bottom 8-well chamber slides using a Fluoview FV3000 confocal microscope equipped with 60× oil immersion objectives, as above. Condensates were bleached using a laser intensity of 30% at 561 nm. Recovery was recorded for every second for a total of 180 s after bleaching. Analysis of the recovery curves was carried out with cellSens software (Olympus).

### Statistical analysis

For all quantified data, mean ± standard deviation (SD) was presented. Statistical significance between two experimental groups was indicated by an asterisk and comparisons were made using the Student’s *t* test. *P* values less than 0.05 were considered significant.

## Supplementary Information

**Additional file 1.**

## Data Availability

The datasets supporting the conclusion of this article are included within the article.

## Abbreviations

ATP: Adenosine triphosphate
CD: Circular dichroism
DFC: Dense fibrillar component
FBL: Fibrillarin
FRAP: Fluorescence recovery after photobleaching
GC: Granular component
GTP: Guanosine triphosphate
IDP: Intrinsically disordered protein
IDR: Intrinsically disordered region
JAGs: Juvenility-associated genes
KO: Knockout
NMR: Nuclear magnetic resonance
PCM: Pericentriolar matrix
PEG: Polyethylene glycol
RPE: Retinal pigment epithelium
WT: Wild type
