# Supplementary material for: Bex1 is essential for ciliogenesis and harbours biomolecular condensate-forming capacity
Source: BMC Biol. 2022 Feb 10;20:42. doi: 10.1186/s12915-022-01246-x (PMC8830175; doi:10.1186/s12915-022-01246-x)
Supplement: Supplementary file 1 — Additional file 1. [file 12915_2022_1246_MOESM1_ESM.pdf]

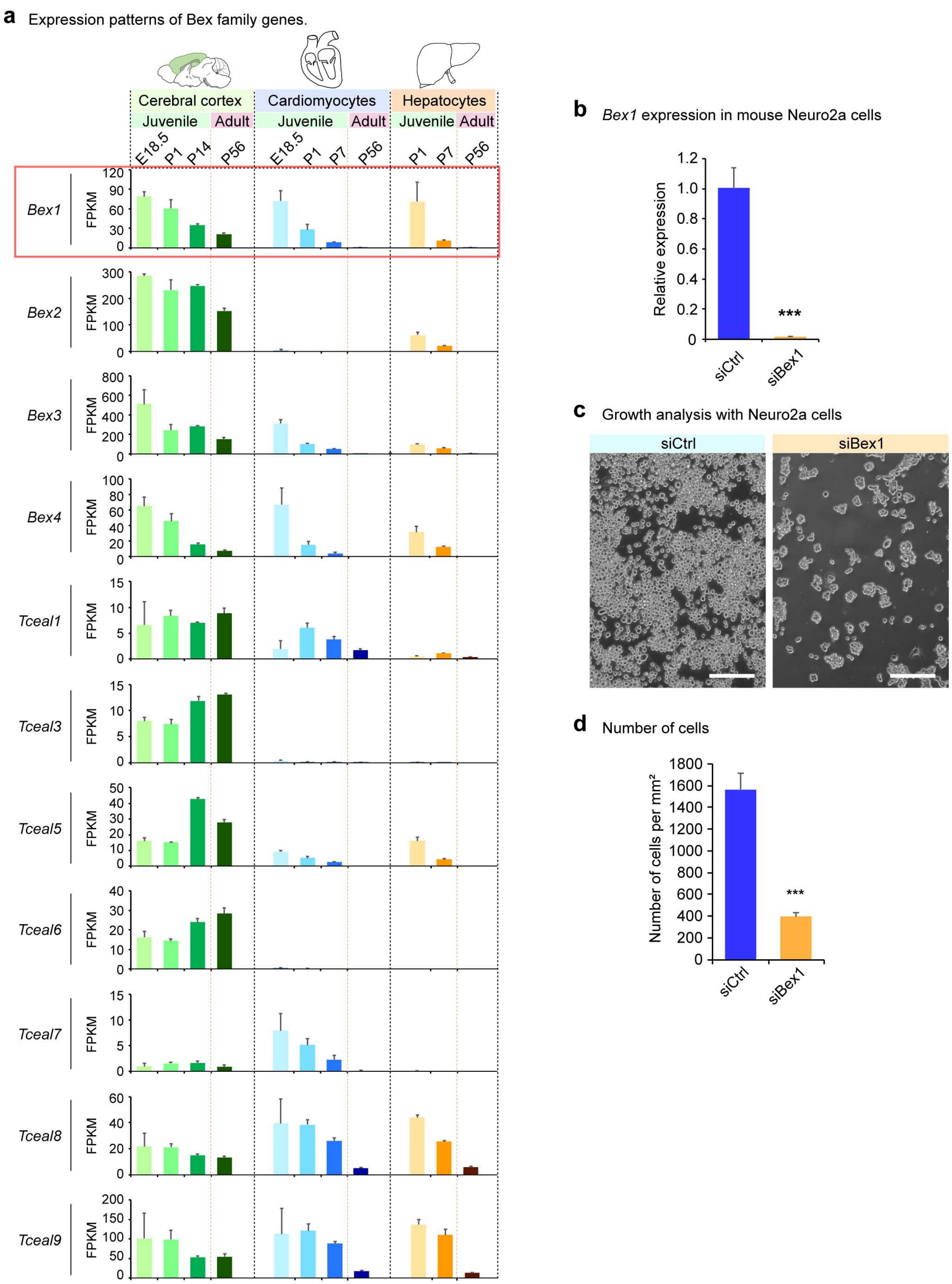

Fig. S1. *Bex1* is expressed in juvenile organs and essential for cellular growth.

(a) The expression levels (fragments per kilobase of exon per million mapped fragments, FPKM) of the 11 Bex family genes in the cerebral cortex, isolated cardiomyocytes and isolated hepatocytes of juvenile (E18.5, P1, P7 and p14) and adult (P56) mice.

(b) qPCR analysis of *Bex1* in mouse Neuro2a cells 48 hours after transfection of control siRNA or *Bex1* siRNA. Data were normalized to *Tubb5*.

(c) Phase contrast images of Neuro2a cells 72 hours after transfection of control siRNA or *Bex1* siRNA. Cellular growth was suppressed by *Bex1* depletion. Scale bar = 250 µm.

(d) Number of cells per field 72 hours after transfection with control siRNA or *Bex1* siRNA.

*** $p < 0.001$; Student's *t*-test. The data are presented as the means ± standard deviations.

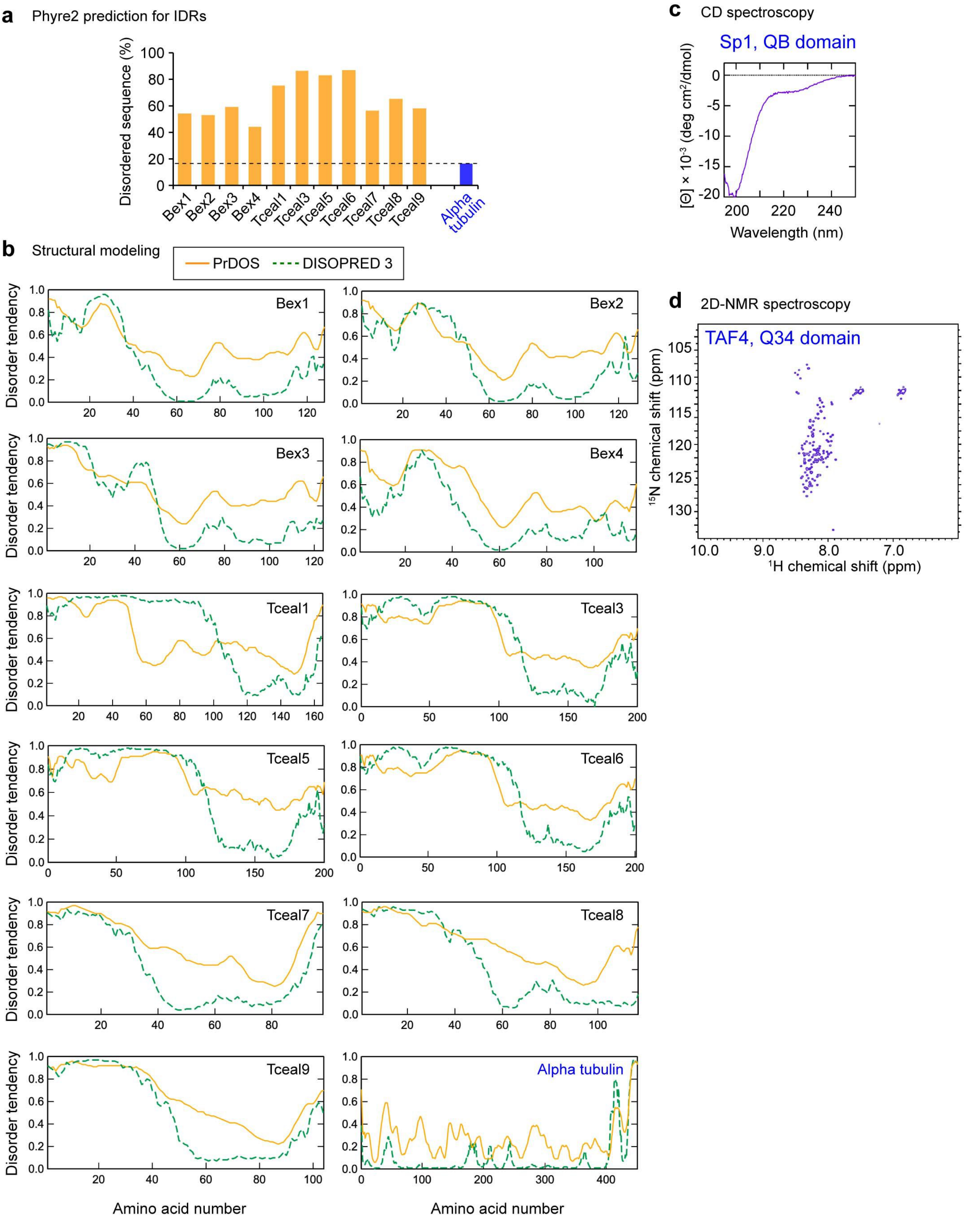

Fig. S2. Modeling of the physical property.

(a) Phyre2 analysis of the Bex family proteins. The result with alpha tubulin (α-tubulin) was shown for comparison. Bex family protein contained longer disordered sequences compared to alpha-tubulin (blue).

(b) All Bex family proteins contained intrinsically disordered regions as modeled by PrDOS (orange solid line) and DISOPRED3 (green dotted line) algorithms. The result with alpha tubulin (α-tubulin) was shown as an example of a structured protein (lower right).

(c) CD spectrum of the recombinant Sp1 QB domain at 10°C. A typical random coil CD spectrum was detected. Θ, molar ellipticity.

(d) 2D-NMR spectrum of the ^{15}N-labeled recombinant TAF4 Q34 domain. The ^{1}H-^{15}N HSQC spectrum was measured at 37°C. The spectral data from Hibino et al. (2017) was reanalysed.

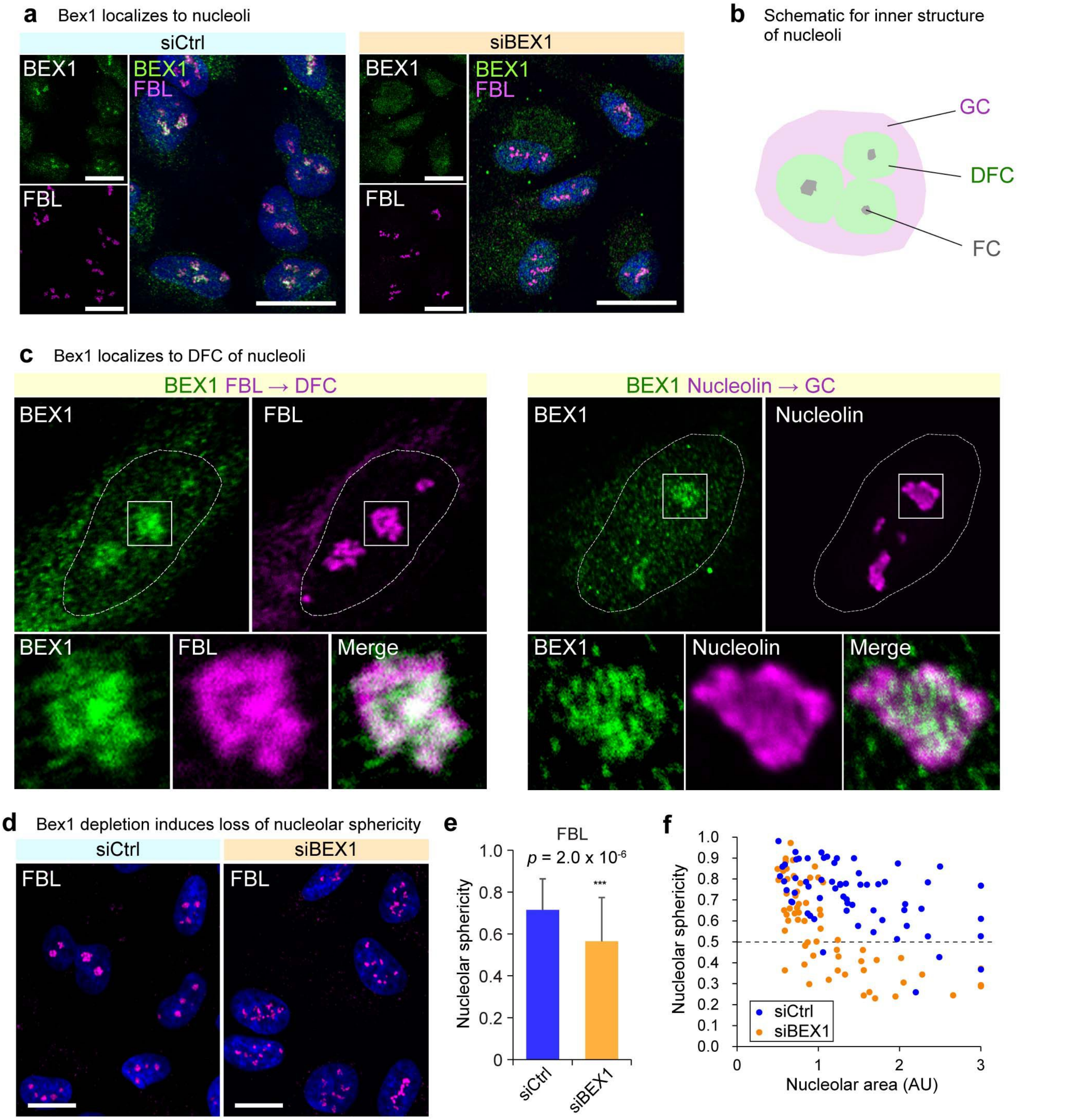

Fig. S3. Bex1 contributes to the maintenance of DFC morphology in nucleoli.

(a) Immunostaining of BEX1 in ARPE19 cells at the low cell density. The ARPE19 cells were transfected with control siRNA or *Bex1* siRNA and incubated for 72 hrs prior to immunostaining. Nucleoli and nuclei were counterstained with fibrillarin (FBL) antibody and DAPI, respectively. Scale bar = 25 μm.

(b) Schematic representation of the nucleolar inner structure that consists of granular component (GC), dense fibrillar component (DFC) and fibrillar center (FC).

(c) Immunostaining to examine the colocalization of BEX1 and the nucleolar protein FBL and Nucleolin. The nuclear shape was depicted by the dot line. Insets show the magnified views of nucleoli. Bex1 showed better colocalization to FBL compared to Nucleolin.

(d) Immunostaining of FBL in ARPE19 cells transfected with control siRNA or *BEX1* siRNA followed by culture for 72 hrs. Nuclei were counterstained with DAPI. Scale bar = 10 μm.

(e) Nucleolar sphericity assessed by calculating the circularity of nucleoli stained with FBL antibody. *** $p = 2.0 \times 10^{-6}$; Student' s *t*-test. The data are presented as the means ± standard deviations.

(f) Scatter plot for the nucleolar sphericity assessed by FBL immunostaining in ARPE19 cells transfected with control siRNA (blue dots) or *BEX1* siRNA (orange dots).

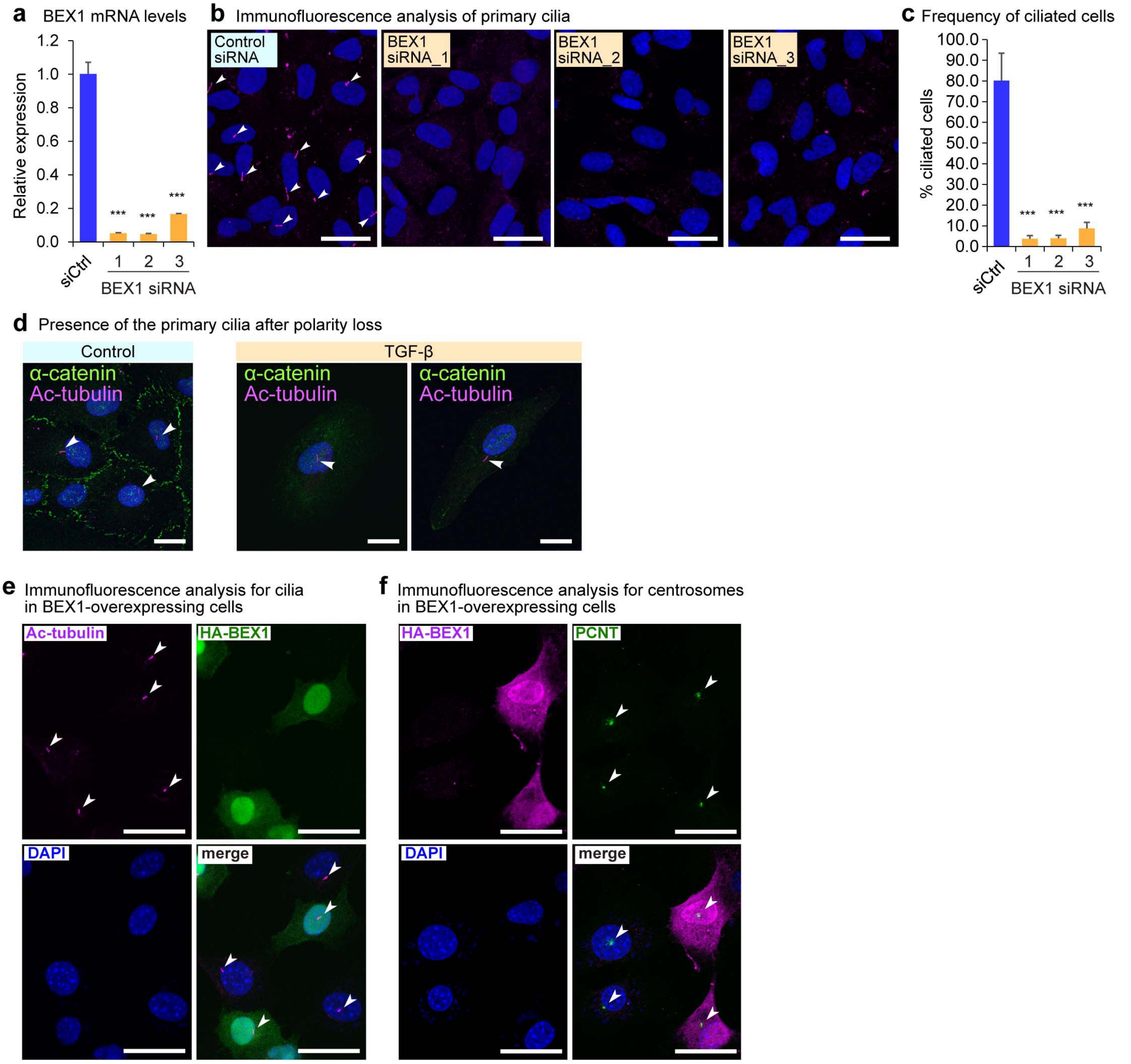

Fig. S4. Analysis of ciliogenesis in cultured cells.

(a) qPCR analysis of BEX1 in human ARPE19 cells 48 hours after transfection of control siRNA or BEX1 siRNAs. Data were normalized to TUBB.

(b) Immunofluorescence analysis for the primary cilia in ARPE19 cells transfected with control siRNA or the indicated BEX1 siRNA. Cilia formation was abrogated by the BEX1 depletion. Nuclei were stained with DAPI. Scale bar = 25 μm.

(c) Frequency of ciliated cells after transfection with control siRNA or BEX1 siRNA. The number of ciliated cells was counted in the experiment (b).

(d) Analysis of ciliogenesis in cells losing cell polarity. ARPE19 cells were treated 10 ng/ml TGF-β for 48 hrs to induce the loss of cell polarity. Without TGF-β treatment, cells harboured epithelial property and express junctional α-catenin. After TGF-β treatment, cells lost cell polarity exhibiting scattering and loss of junctional α-catenin. Ac-tubulin-stained cilia are formed in the TGF-β-treated cells. Scale bar = 10 μm.

(e) Influences of Bex1 forced expression on ciliogenesis were assessed in NIH3T3 cells. Immunostaining for Ac-tubulin and HA antigen were performed in NIH3T3 cells 48 hrs after transfection of HA-BEX1. Scale bar = 25 μm.

(f) Influences of Bex1 forced expression on PCNT localization to the centrosome were assessed in NIH3T3 cells. Immunostaining for PCNT and HA antigen were performed in NIH3T3 cells 48 hrs after transfection of HA-BEX1. Scale bar = 25 μm. *** $p < 0.001$; Student' s t-test. The data are presented as the means ± standard deviations.

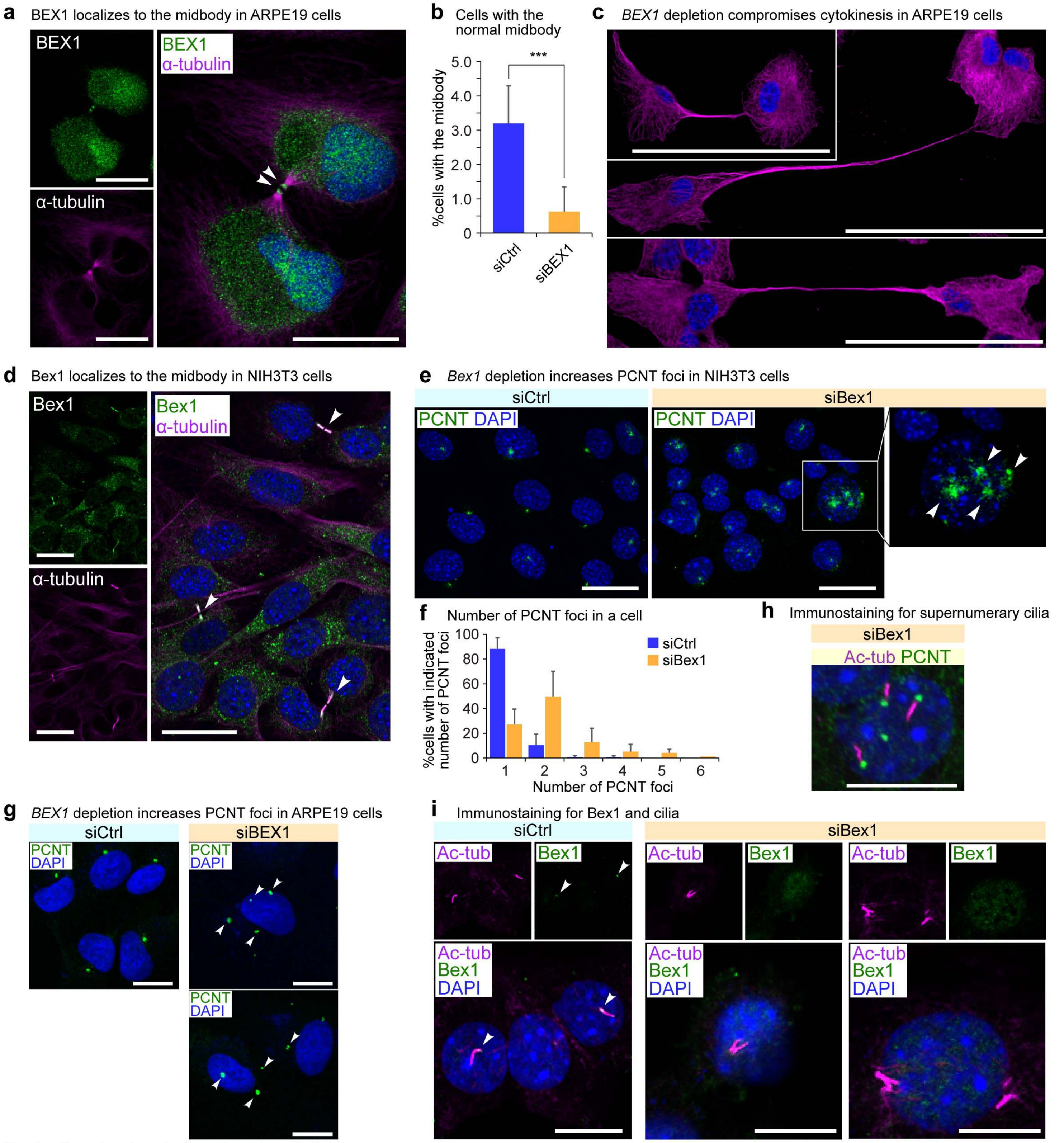

Fig. S5. Functional analysis of Bex1 in cell division

(a) Immunostaining of BEX1 and α-tubulin in ARPE19 cells undergoing cell division. Arrowhead indicates the localization of BEX1 to the midbody. Nuclei were stained with DAPI. Scale bar = 25 µm. **(b)** Frequency (%) of cells with the midbody. ARPE19 cells were transfected with control siRNA or BEX1 siRNA and cultured for 48 hrs prior to immunostaining with α-tubulin. The number of cells harbouring the midbody with dense tubulin organization were count. **(c)** Representative images of the BEX1-depleted cells undergoing incomplete abscission. Scale bar = 100 µm. **(d)** Immunostaining of Bex1 and α-tubulin in NIH3T3 cells. Bex1 localized to midbodies. The antibody responsive to mouse Bex1 we prepared was used. Nuclei were stained with DAPI. Scale bar = 25 µm. **(e)** Immunostaining of Pericentrin (PCNT) in NIH3T3 cells. Cells were transfected with control siRNA or Bex1 siRNA and cultured for 48 hrs. Bex1-depleted cells exhibited the increased PCNT signals corresponding to centrosomes and the nuclei with increased size or aberrant shape. Inset shows the cell with increased PCNT foci. Scale bar = 25 µm. **(f)** Frequency (%) of cells with indicated number of PCNT foci. Most of control knockdown cells showed a single PCNT focus whereas majority of Bex1-depleted cells harboured multiple PCNT foci. **(g)** Immunostaining of Pericentrin (PCNT) in ARPE19 cells. Cells were transfected with control siRNA or BEX1 siRNA and cultured for 48 hrs. BEX1-depleted cells exhibited the increased PCNT signals. Scale bar = 10 µm. **(h)** A representative image of supernumerary Bex1-depleted NIH3T3 cell with multiple cilia stained with acetylated tubulin and PCNT-labelled basal bodies. Scale bar = 10 µm.
(i) Co-immunostaining of Bex1 and supernumerary cilia. Bex1 and Ac-tubulin were co-immunostained in NIH3T3 cells transfected with Ctrl siRNA or Bex1 siRNA to induce generation of supernumerary cilia. Bex1 signals were lost in the bases of supernumerary cilia. Scale bar = 10 µm.

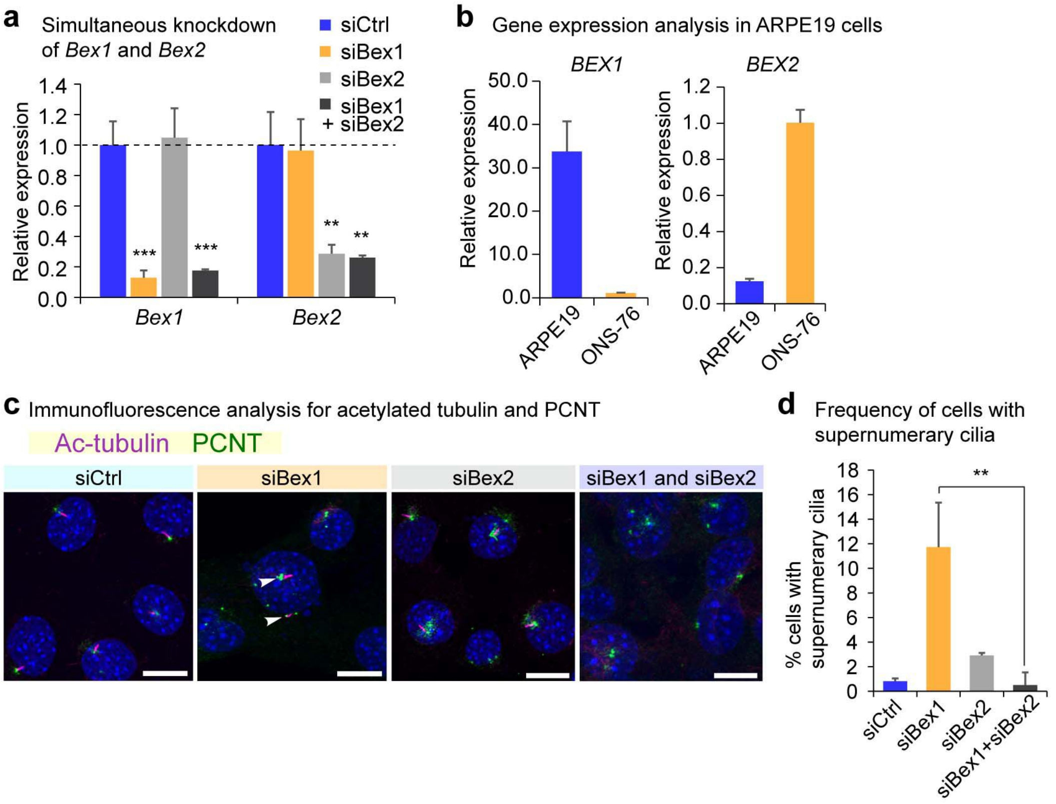

Fig. S6. *Bex1* depletion leads to the formation of supernumerary cilia dependent on *Bex2*.

(a) qPCR gene expression analysis for *Bex1* and *Bex2* in NIH3T3 cells transfected with the indicated siRNAs. Cells were cultured 72 hrs prior to RNA extraction. Expression levels were normalized to *Tubb5*.

(b) qPCR gene expression analysis for *BEX1* and *BEX2* in ARPE19 cells. Expression levels were normalized to *TUBB*.

(c) Immunostaining of acetylated tubulin and PCNT for assessment of supernumerary cilia formation. NIH3T3 cells were transfected with indicated siRNAs and cultured for 72 hrs prior to immunofluorescence analysis. Arrowheads indicate supernumerary cilia found in *Bex1*-depleted cells. Scale bar = 10 µm.

(d) Frequency of cells with supernumerary cilia. Number of cells with supernumerary cilia was counted among the total cells.

** $p < 0.01$, *** $p < 0.001$; Student' s *t*-test. The data are presented as the means ± standard deviations.

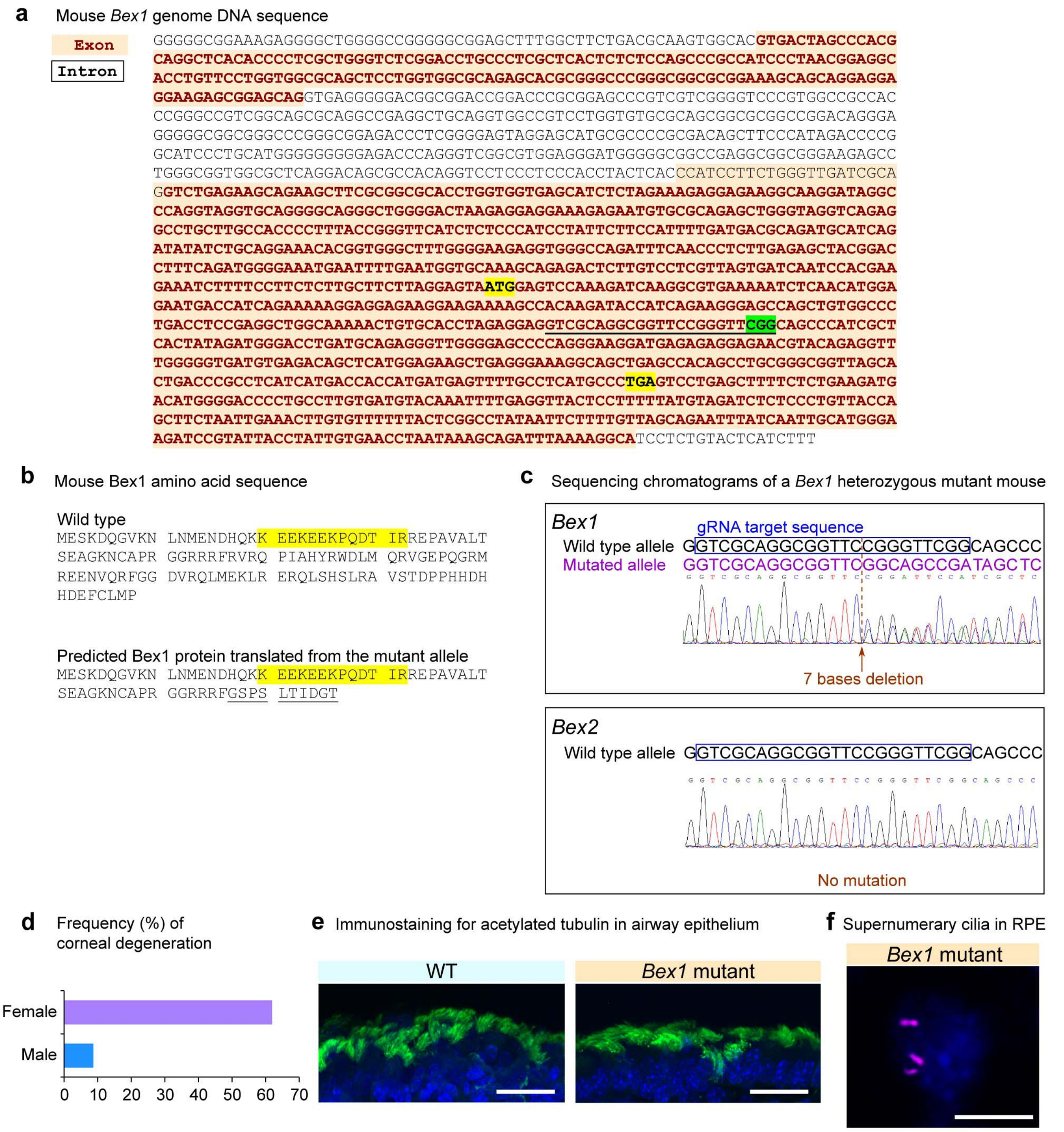

Fig. S7. *Bex1* Δ7 allele and sex difference of *Bex1* mutant mice.

(a) The genome sequence of the mouse *Bex1* locus. Exons were highlighted by orange color. Start (ATG) and stop (TGA) codons were highlighted by yellow. The guide RNA-target sequence was underlined. The protospacer adjacent motif (PAM) sequence was indicated by green.

(b) Predicted amino acid sequence of the protein translated from wild-type and Δ7 mutant *Bex1*. Highlighted is the epitope used to develop the antibody against mouse Bex1. Underlined is the mutated sequence that terminates because of the stop codon.

(c) The sequencing chromatograms obtained with a *Bex1* heterozygous mutant mouse. The gRNA target sequence was shown in blue. The *Bex1* mutant strain did not have a mutation in *Bex2* harboring the identical gRNA target sequence.

(d) Frequency of the corneal degeneration in male and female *Bex1* KO mice. WT mice did not present corneal degeneration (not shown).

(e) Immunostaining of acetylated tubulin in airway epithelium in wild-type (WT) and *Bex1* mutant mice. The presence of motile cilia was assessed in airway epithelium of WT and *Bex1* mutant mice. The motile cilia were not lost in *Bex1* mutant mice. Nuclei were counterstained with DAPI. Scale bar = 25 μm.

(f) Representative image of the supernumerary cilia in RPE of Bex1 mutant mice. Scale bar = 5 μm.

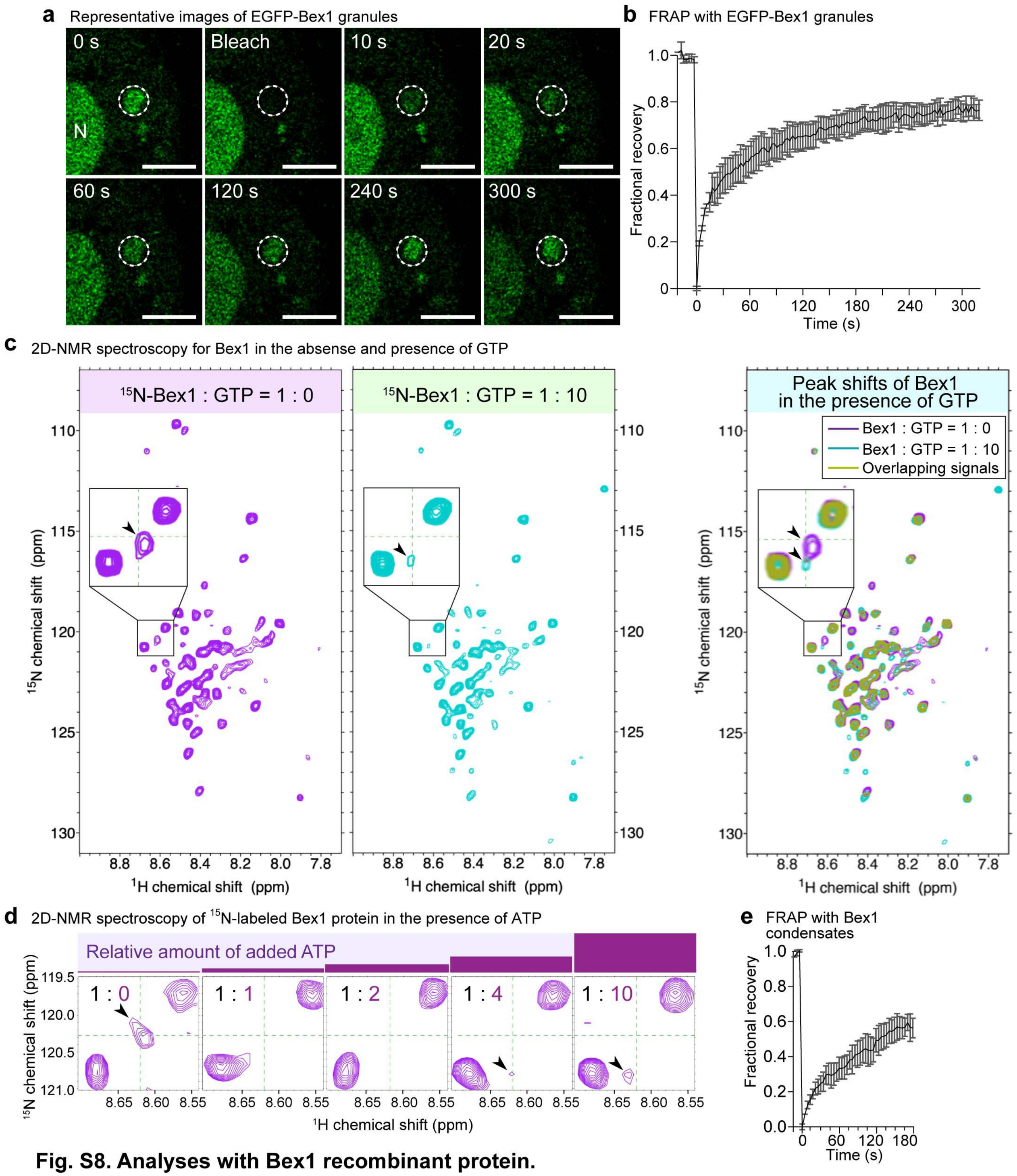

Fig. S8. Analyses with Bex1 recombinant protein.

(a) An illustrative example of EGFP-Bex1 fluorescence recovery after photobleaching (FRAP), which was used to examine the dynamic nature of Bex1 granules formed in cells. Dashed line indicates the bleached area. N, nucleus. Scale bar = 5 µm.

(b) Plot showing the time course of the recovery after photobleaching EGFP-Bex1 granules. Data are presented as mean ± s.d. (n = 5).

(c) Left, the 2D-NMR spectra of ^{15}N-labeled recombinant Bex1 protein in the absence (molar ratio of ^{15}N-Bex1 : GTP = 1 : 0) and presence (^{15}N-Bex1 : GTP = 1 : 10) of GTP. The ^{1}H-^{15}N HSQC spectra were measured at pH 7.3 and 25°C. Right, the merged image of the spectra in the absence and presence of GTP. The overlapping peaks were shown in green. The peaks without the overlap indicate the peaks were shifted by the addition of GTP. The inset in the left panel indicated the area that were used for the calculation of the dissociation constant (*Kd*) of Bex1 and GTP in **Fig. 7c,d**.

(d) The 2D-NMR spectroscopy with ^{15}N-labeled recombinant Bex1 protein in the presence of indicated molar ratios of Bex1 to adenosine triphosphate (ATP). The ^{1}H-^{15}N HSQC spectra were measured at pH 7.3 and 25°C.

(e) Plot showing the time course of the recovery after photobleaching Bex1 condensates. Data are presented as mean ± s.d. (n = 5).

Figure 5a

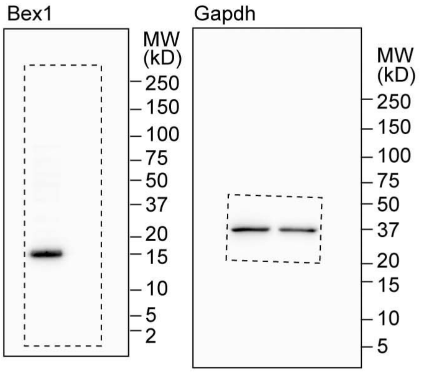

Fig. S9. Images of the uncropped blots
